# Survival of cancer cells following taxol-induced apoptosis is supported by ZEB1-mediated ferroptosis sensitivity

**DOI:** 10.1038/s41419-026-09242-7

**Published:** 2026-09-02

**Authors:** Rachel Hausman, Wells S. Brown, Shannon J. Awrey, Hossein Tavakoli, Denise J. Montell, Paul C. McDonald, Shoukat Dedhar

**Affiliations:** 1 Department of Basic and Translational Research, BC Cancer Research Institute, Vancouver, BC Canada; 2 Faculty of Medicine, University of British Columbia, Vancouver, BC Canada; 3 Department of Molecular, Cellular, and Developmental Biology, University of California, Santa Barbara, CA USA; 4 Department of Biochemistry and Molecular Biology, University of British Columbia, Vancouver, BC Canada

**Keywords:** Transcription factors, Breast cancer, Apoptosis, Metabolomics

## Abstract

Chemotherapy remains the mainstay treatment of many solid cancers; however, resistance and recurrence compromise patient outcomes. We investigated chemotherapy resistance mechanisms in Triple Negative Breast Cancer (TNBC) by examining cells that survive chemotherapy-induced apoptotic caspase activation. Using a biosensor that permanently labels cells surviving executioner caspase activation with GFP, we identified a GFP+ population that survives transient paclitaxel treatment. These GFP+ cells display a stem-like invasive phenotype, altered mitochondrial metabolism, and increased drug resistance. Metabolomics profiling identified defects in TCA cycle metabolites and accumulation of polyunsaturated fatty acids (PUFAs). Mechanistically, surviving GFP+ cells exhibited Epithelial to Mesenchymal Transition (EMT) and ZEB1-regulated repression of GPX4 expression, resulting in increased sensitivity to ferroptotic cell death. Consistent with this, transforming growth factor- beta induced EMT also suppressed GPX4 and increased ferroptosis sensitivity in Normal Mouse Mammary epithelial cells. ZEB1 knockdown strongly reduced the GFP+ surviving fraction, identifying ZEB1 as a key regulator of apoptotic survival. Supplementation with arachidonic acid restored survival in ZEB1 knockdown cells, while the ferroptosis-protective drug, Ferrostatin-1, prevented the accumulation of GFP+ survivors, demonstrating that ferroptosis sensitivity is required for the survival of TNBC cells from chemotherapy-induced apoptosis. Together, these findings reveal a previously unrecognized link between ferroptosis and apoptosis and identify potential biomarkers and therapeutic vulnerabilities in a subset of chemotherapy-resistant TNBC.

## Introduction

In today’s landscape of cancer therapeutics, recurrent tumors present a barrier to complete eradication of the disease in patients. Adaptive responses by primary tumor cells to chemotherapy-induced apoptosis can result in the incomplete killing of cancer cells, leading to recurrence post-chemotherapy [1, 2]. Therefore, there is an urgent need for a better understanding of the mechanisms by which cancer cells survive chemotherapy to facilitate the development of novel therapeutic strategies that will prevent the survival of such cells.

The majority of chemotherapy agents kill cancer cells by apoptosis, and resistance to the induction of apoptosis by executioner caspases has been found to contribute to drug resistance [3]. Apoptosis is a form of programmed cell death in which a stimulus triggers mitochondrial outer membrane permeabilization (MOMP), leading to the subsequent release of cytochrome C, which binds with Apaf-1 and forms the apoptosome, ultimately resulting in executioner caspase activation [4, 5]. It was previously thought that MOMP and subsequent release of executioner caspases into the extracellular space was the “point of no return” from apoptotic death [6–8]. However, in a process termed anastasis, cells can recover from transient executioner caspase activation upon removal of the apoptosis-inducing agent [6, 9]. Cancer cells have been shown to survive drug-induced apoptosis and cellular stress via anastasis, which may act as an adaptive mechanism for the survival of a sub-population of tumor cells, leading to drug resistance [4, 6, 9–15]. Cancer cells have also been shown to overcome apoptosis when levels of cytochrome c release are insufficient to induce executioner caspase activation, leading to the generation of drug-tolerant persister cells (DTPs) [16]. In addition, cells have been reported to overcome apoptosis in an event referred to as “minority MOMP”, whereby cells activate executioner caspases at levels insufficient to induce apoptosis [17]. Anastatic cells differ from these mechanisms since these cells experience executioner caspase activation upon cytochrome c release [6].

Here, using a biosensor (CasExpress) that uniquely identifies cells that survive following executioner caspase activation, we demonstrate a critical role for ZEB1, an effector of EMT, not only in the acquisition of anastasis in response to paclitaxel-mediated apoptosis, but also in lipid and metabolic rewiring of surviving, anastatic cells. Our findings demonstrate an essential role of ZEB1 in the regulation of expression of Glutathione Peroxidase 4 (GPX4), a key regulator of ferroptosis sensitivity, and demonstrate that ZEB1-mediated downregulation of GPX4 is a critical event in the increased sensitivity of chemotherapy-induced apoptosis-surviving Triple Negative Breast Cancer (TNBC) cells to ferroptosis, a mechanism that appears to be essential for anastatic cell survival.

## Results

### Identification and isolation of triple-negative breast cancer cells surviving paclitaxel-induced caspase activation

**Fig. 1:**
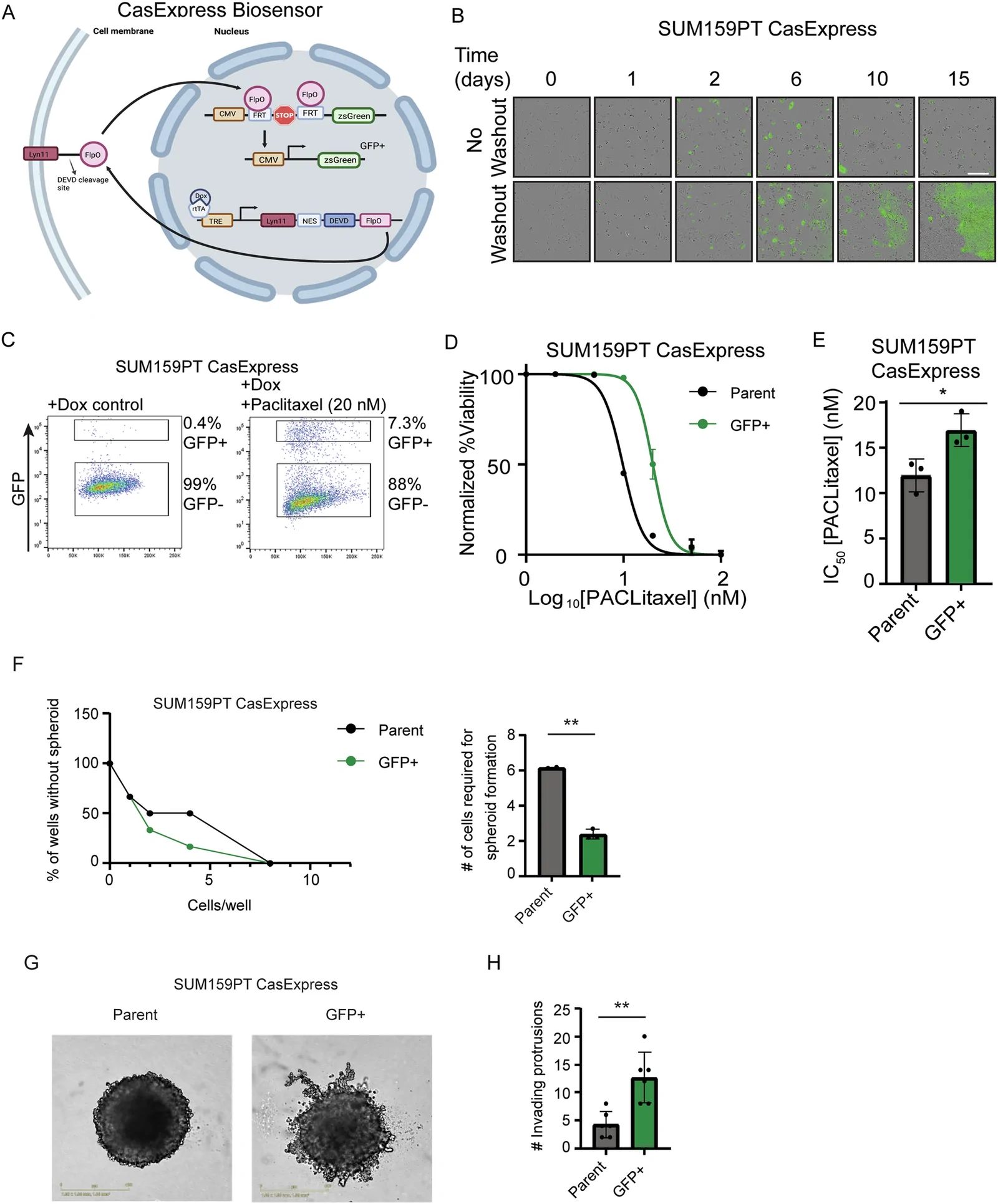
Identification and isolation of SUM159PT GFP+ cells. **A** Schematic representing the CasExpress Biosensor. **B** Incucyte time-lapse imaging of SUM159PT CasExpress cells treated with 1ug/mL doxycycline and 20 nM paclitaxel followed by a recovery period of 14 days. Scale bar in the top right corner indicates 300 µm. **C** FACS-based sorting of SUM159PT CasExpress GFP+ cells treated as described in (**B**). GFP+ cells were collected to establish the SUM159PT CasExpress GFP+ cell line. **D** Cytotoxicity curve for SUM159PT CasExpress cells treated with 0-200 nM paclitaxel. IC50 values were calculated using Nonlinear regression analysis in Prism. **E** IC_50_ values for SUM159PT cells treated with 0-200 nM paclitaxel for 24 h (*n* = 4). Cell death was quantified using Sytox Orange stain via Incucyte imaging and normalized using the Incucyte Cell-by-Cell function. (*N* = 3). **F** Sphere Limiting Dilution Assay was performed by growing Mammospheres in Mammocult media for 7 days to assess the ability of the cells to form Mammospheres as a functional assay for stemness. (*n* = 8; *N* = 3). **G** Images of SUM159PT Mammospheres (*N* = 3). **H** Quantification of the number of invasive protrusions (*n* = 6; *N* = 3).

To study anastasis as a mechanism for survival of TNBC cells from chemotherapy treatment, we first sought to identify and isolate a population of these cells surviving chemotherapy-induced apoptotic caspase activation using the CasExpress biosensor (Fig. 1A). Chemotherapy remains the mainstay of treatment for TNBC, which has a high rate of drug resistance and tumor recurrence leading to metastases to the brain and lung, and an overall worse prognosis, highlighting a need for alternative therapeutic strategies to treat TNBCs [18]. The CasExpress biosensor labels anastatic cells by permanently expressing GFP in those that have activated executioner caspases, even transiently (Fig. 1A) [19]. This biosensor contains a plasmid encoding a Lyn11 domain for insertion into the plasma membrane and a caspase-specific DEVD domain, which is cleaved during executioner caspase activation in the cell (Fig. 1A). This cleavage results in the translocation of Flp recombinase into the nucleus where it recognizes and binds to FRT sequences, resulting in the excision of a stop codon that allows for permanent GFP expression (Fig. 1A). CasExpress was transduced into SUM159PT TNBC cells which were subsequently treated with 20 nM paclitaxel for 24 h, after which the chemotherapy was removed and cells were observed for the presence of GFP+ (anastatic) cells by Incucyte time-lapse imaging during a recovery period of 14 days (Fig. 1B). Following paclitaxel removal, time-dependent accumulation of GFP+ cells was observed, demonstrating the ability of SUM159PT TNBCs to survive paclitaxel-induced executioner caspase activation by undergoing anastasis (Fig. 1B).

We next sought to establish the anastatic CasExpress GFP + TNBC cell lines. SUM159PT, MDA-MB468, Hs578t, and HCC1806 CasExpress cell lines were exposed to paclitaxel for 24 h, after which the drug was removed, and cells were allowed to recover for 14 days. Subsequently, cells were FACS-sorted based on GFP expression, and GFP+ cells were collected and subcultured to establish “anastatic” cell lines (Fig. 1C for SUM159PT cells). As shown in Supplementary Fig. 1A, these anastatic cells were still capable of in vitro colony formation following recovery from paclitaxel treatment. We next validated the expression of GFP in the collected anastatic population using PCR by measuring the expression of the zsGreen construct generated using the CasExpress system [11]. The 864 bp “zsGreen + ” plasmid corresponding to the CasExpress GFP+ phenotype (Supplementary Fig. 1B) was present in the cells [11]. Collectively, these data demonstrate that some TNBC cells survive paclitaxel-induced executioner caspase activation, and that permanent GFP expression following caspase activation enables their identification and isolation for downstream analyses.

Since recurrent tumors are known to be chemoresistant, we also evaluated these cells for sensitivity to paclitaxel upon rechallenge [1, 20]. Anastatic cells demonstrated increased resistance to paclitaxel treatment compared to the parental cells (Fig. 1D, E and Supplementary Fig. 2), in addition to decreased levels of executioner caspase-3 in the SUM159PT anastatic cells (Supplementary Fig. 1C), demonstrating that anastatic TNBC cells develop resistance to chemotherapy.

It was previously shown that cells that have undergone stress-induced anastasis exhibit plasticity associated with epithelial to mesenchymal transition (EMT) and associated stem cell properties [6]. To determine whether chemotherapy-treated cancer cells also exhibit this phenotype, we grew mammospheres derived from SUM159PT CasExpress Parental and GFP+ cell lines in Stem Cell Mammocult media and performed a sphere limiting dilution assay (SLDA). This revealed that, compared to the parental cells, the anastatic GFP+ cells have an increased sphere-forming capability, indicative of an enhanced stem cell phenotype (Fig. 1F). Imaging of these mammospheres revealed a striking invasive phenotype in the GFP+ cells, with many invasive protrusions (Fig. 1G, H).

### Breast cancer cells surviving apoptotic caspase activation exhibit evidence of mitochondrial rewiring

**Fig. 2:**
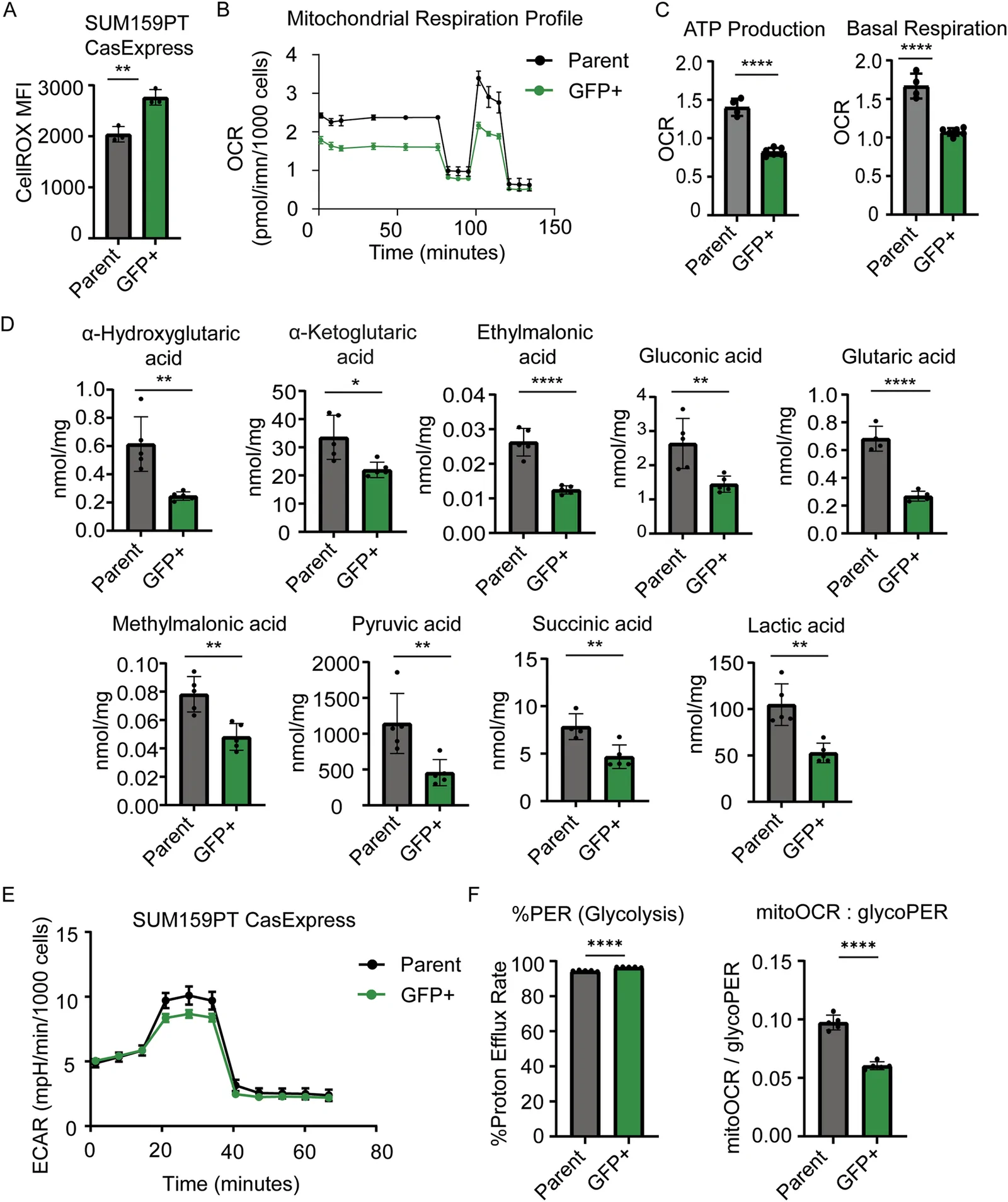
Characterization of mitochondrial function in SUM159PT CasExpress GFP+ cells. **A** Flow cytometry data measuring the total ROS in SUM159PT CasExpress cells using CellROX staining (*n* = 3). (*N* = 2). **B** Data demonstrating the mitochondrial respiration profile using the Agilent Seahorse Mito Stress Test. The graph shows the oxygen consumption rate of cells for the duration of the Seahorse Mito Stress Test. OCR is a measurement of mitochondrial respiration. (*N* = 2). **C** Quantification of the indicated parameters determined from the Agilent Seahorse Mito Stress Test. Data were quantified from the oxygen consumption rate using the Agilent Seahorse software. (*N* = 2). **D** Concentrations of TCA cycle organic acids acquired via UPLC-MRM/MS metabolomics analysis. Significance was determined using the unpaired t-test assuming a Gaussian distribution. Outliers were identified using Grubbs’ test (*n* = 5). **E** Data demonstrating the extracellular acidification rate (ECAR) of the indicated cell line for the duration of the Seahorse Glycolytic Rate test (*n* = 5). ECAR is a measurement of glycolytic activity. (*N* = 3). **F** Quantification of the indicated parameters determined from the Agilent Seahorse Glycolytic Rate assay Test (*n* = 5). Data were quantified from the extracellular acidification rate using the Agilent Seahorse software. (*N* = 3). Unless otherwise indicated, error bars represent the standard deviation of the mean.

Mitochondria play a crucial role in the apoptotic cascade, with MOMP being a requirement for the release of cytochrome c and subsequent activation of executioner caspases [5]. Because cellular stress is associated with impaired function of the electron transport chain (ETC) and increased cellular reactive oxygen species (ROS), we first evaluated these cells for their ROS content using the CellROX stain via flow cytometry [21, 22]. As expected, the SUM159PT GFP+ cells demonstrated an increase in total ROS (Fig. 2A). Next, we tested whether the SUM159PT anastatic cells exhibit metabolic rewiring of the mitochondria. We first assessed mitochondrial function using the Seahorse Mito Stress Test, which revealed that anastatic SUM159PT cells have impaired oxidative phosphorylation compared to the parental cell line (Fig. 2B, C and Supplementary Fig. 3). Due to the deficiencies seen in the mitochondrial ETC, we next sought to evaluate for any changes in the tricarboxylic acid (TCA) cycle using MRM/MS metabolomics analysis in collaboration with the University of Victoria Proteomics and Metabolomics Centre. SUM159PT anastatic cells showed significant deficiencies in numerous metabolites associated with the TCA cycle (Fig. 2D). Finally, we evaluated cells for glycolytic function using the Seahorse Glycolytic Rate Assay. This revealed the SUM159PT anastatic cells to be more reliant on glycolysis when compared to parental controls, as shown by the significant increase in the percentage of glycolytic proton efflux rate and decrease in the mitoOCR/glycoPER ratio, which is indicative of an increased reliance on glycolysis for ATP production (Fig. 2E, F). These findings suggest that the anastatic SUM159PT cells undergo metabolic rewiring following MOMP and cytochrome c release to compensate for the resulting decrease in mitochondrial function. These findings also reveal glycolysis, specifically pyruvate and lactate availability, as a potentially targetable vulnerability in the anastatic SUM159PT cells.

### Breast cancer cells surviving apoptotic caspase activation are highly susceptible to ferroptosis

**Fig. 3:**
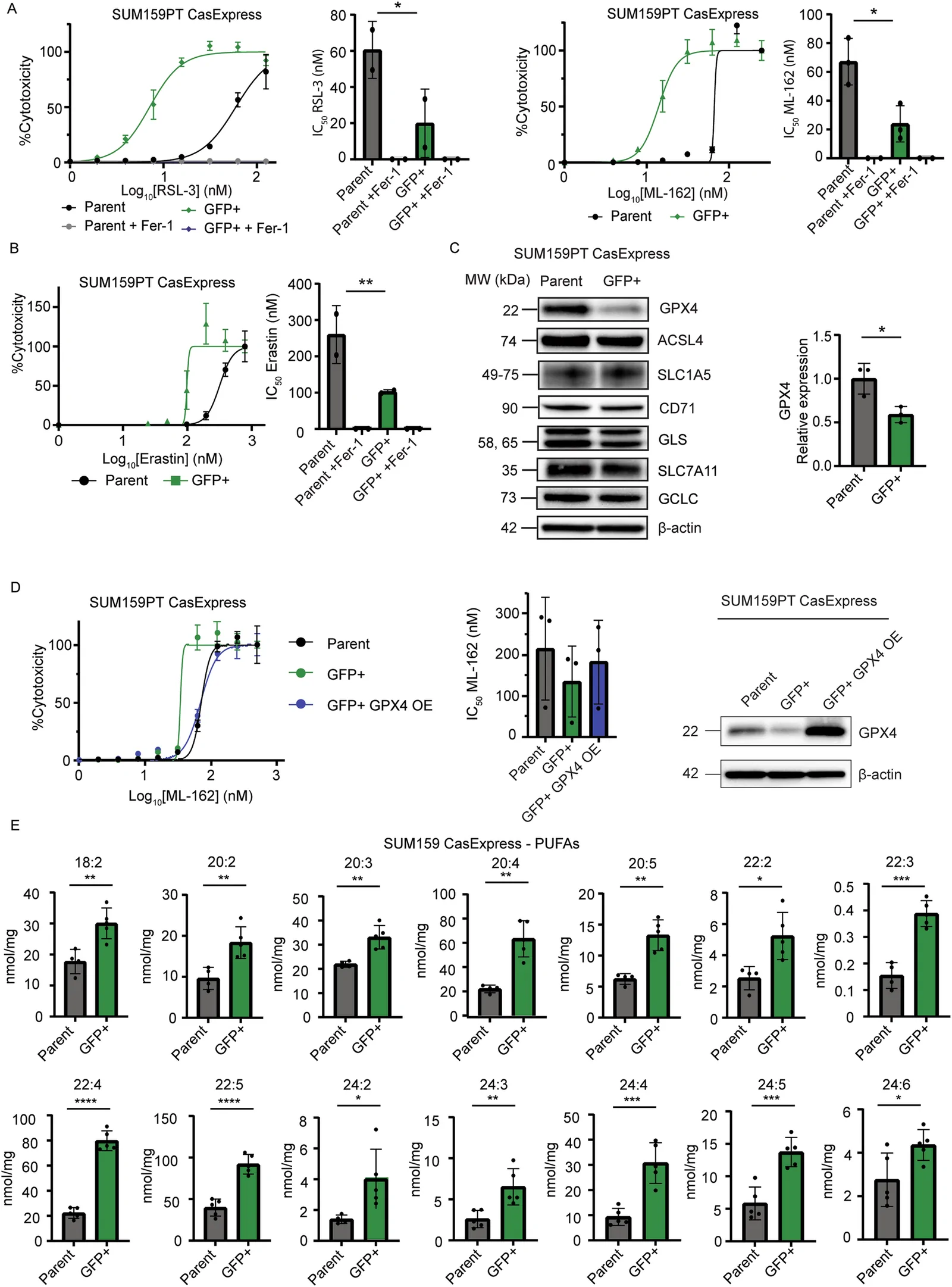
Characterization of alterations in the SLC7A11-GSH-GPX4 axis and sensitivity to ferroptosis in SUM159PT CasExpress GFP+ cells. **A** Cytotoxicity curve and corresponding IC50 values for SUM159PT cells treated with 0-125 nM RSL-3 and 2 μM ferrostatin-1 for 72 h. Cell death was quantified using Sytox Orange stain via Incucyte imaging and normalized using the Incucyte Cell-by-Cell function (Left). Cytotoxicity curve and corresponding IC50 values for SUM159PT cells treated with 0-250nM ML-162 for 72 h. Outliers were determined using Grubbs’ test. (*N* = 3). **B** Cytotoxicity curve and corresponding IC50 values for SUM159PT cells treated with 0-20 μM Erastin for 72 h. Measured using Sytox green stain via Incucyte imaging. IC50 values calculated as in (**A**). (*N* = 2). **C** Western blot showing expression of ferroptosis proteins in SUM159PT CasExpress cell lines. Quantified via densitometric analysis in Bio-Rad Image Lab relative to the β-actin loading control. (*N* = 3). **D** Cytotoxicity curve and corresponding IC50 values for SUM159PT cells treated with 0-500nM ML-162 for 72 h. (*N* = 3). Right shows validation of GPX4 overexpression (OE) in the modified cell line. **E** Standardized quantification of Polyunsaturated Fatty Acids. Quantification of organic fatty acids was performed by UPLC-MRM/MS by the University of Victoria Proteomics and Metabolomics Centre. BCA assay was used to standardize all samples (*n* = 5). Significance was determined using *p*-value calculated by the unpaired t-test, assuming a Gaussian distribution. Outliers were identified using the Grubbs Test. Error bars represent the standard deviation of the mean. Nomenclature represents the total number of carbons in the fatty acid tail and the number of double bonds present. Unless otherwise indicated, error bars represent the standard deviation of the mean.

Due to the decreased mitochondrial function seen in SUM159PT cells, and the involvement of mitochondria in ferroptotic cell death, we next evaluated the susceptibility of these cells to ferroptosis by treating them with ferroptosis inducers. We first evaluated sensitivity to ferroptosis using RSL-3 and ML-162, inhibitors of the lipid peroxide detoxification protein GPX4. When treated with either RSL-3 or ML-162, SUM159PT GFP+ cells exhibited an increased sensitivity to both drugs relative to the Parental cells (Fig. 3A). SUM159PT anastatic cells also showed an increased sensitivity to ferroptosis inducer Erastin, an inhibitor of SLC7A11, which imports cystine for the production of GSH, a metabolite used by GPX4 to detoxify lipid peroxides (Fig. 3B). Cell death was rescued by the ferroptosis inhibitor, ferrostatin-1 (Fer-1) (Fig. 3A, B), confirming death due to ferroptosis. A secondary TNBC cell line, MDA-MB-468, also exhibited an increased sensitivity to ferroptosis in the anastatic cells, albeit lower in magnitude, indicating that this is a common feature in TNBCs that survive paclitaxel-induced apoptotic caspase activation (Supplementary Fig. 5B).

To further probe the SLC7A11-GSH-GPX4 axis in SUM159PT anastatic cells, we performed a UPLC-MRM/MS metabolomics analysis on metabolites in this pathway (Supplementary Fig. 4A). While we did not see a change in GSH levels, the total amounts of cysteine, cystine, and glutamic acid are lower in the GFP+ cells, compared to parental control cells (Supplementary Fig. 4A). Interestingly, the levels of oxidized glutathione (GSSG) were significantly lower in the GFP+ cells, concomitant with an increase in the GSH/GSSG ratio (Supplementary Fig. 4A).

Based on the increased sensitivity to ferroptosis in the SUM159PT anastatic cells, together with the various changes seen in the SLC7A11-GSH-GPX4 axis in these cells, we were interested in determining expression levels of ferroptosis effector proteins. These included: CD71 (transferrin receptor) for iron uptake, SLC7A11 (xCT) cysteine transporter, SLC1A5 (ASCT) glutamine transporter, glutamate synthase (GLS), the rate-limiting enzyme for glutathione synthesis (GCLC), ACSL4, which regulates lipid biosynthesis by activating PUFAs, and GPX4, which protects against lipid peroxidation and thus ferroptosis. While there were no significant differences in the expression levels of most of these proteins in the GFP + SUM159PT cells, a striking exception was GPX4, which was expressed at significantly lower levels in both SUM159PT and MDA-MB468 GFP+ cells relative to parental controls (Fig. 3C and Supplementary Fig. 5A). Overexpression of GPX4 in the SUM159PT GFP+ cells restored their resistance to ferroptosis (Fig. 3D). These data suggest that the reduced expression of GPX4 in the GFP+ cells leads to increased vulnerability to ferroptosis, since GPX4 protects cells from ferroptosis by lipid peroxidation and detoxification.

We next wanted to evaluate these cells for changes in levels of polyunsaturated fatty acids (PUFAs). PUFAs are critical for ferroptosis, as these fatty acids are highly susceptible to oxidation and contribute to the lipid peroxide pool within the cell [23]. We analyzed a panel of 18 PUFAs by performing a UPLC-MRM/MS metabolomics analysis, which revealed a significant increase in 14 PUFAs (Fig. 3E and Supplementary Fig. 4B). By contrast, only one of the MUFAs we profiled was significantly altered in the anastatic cells (Supplementary Fig. 4C). This increase in the ratio of PUFAs to MUFAs in anastatic cells compared to the parental cells, paired with the down-regulation of GPX4, is consistent with increased sensitivity to ferroptosis [24].

### ZEB1 regulates sensitivity to ferroptosis in breast cancer cells surviving apoptotic caspase activation

**Fig. 4:**
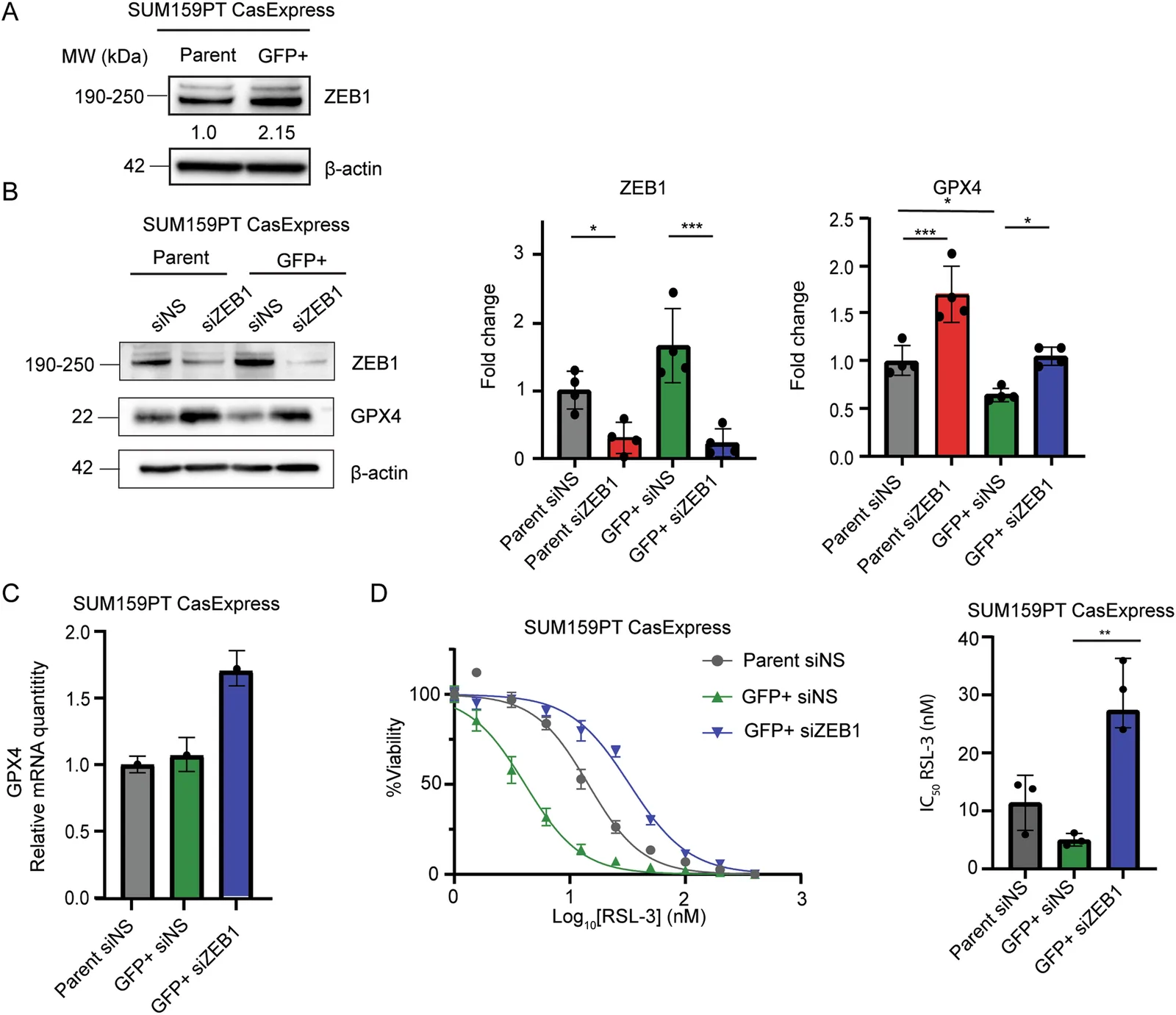
ZEB1 regulates sensitivity to ferroptosis by suppression of GPX4 and regulation of PUFAs. **A** Western blot showing the expression of ZEB1. Β-actin serves as the loading control. Fold changes are indicated below the relevant band and were quantified relative to SUM159PT Parent cells via densitometric analysis in Bio-Rad Image Lab. (*N* = 3). **B** Western blot showing the expression of ZEB1 and GPX4 72 h post-transfection with control or ZEB1-targeting siRNA. Quantification via densitometric analysis in Bio-Rad Image Lab relative to the β-actin loading control in SUM159PT cells seeded at 1.5 × 10^5^ cells per well in a 6-well plate and incubated for 24 h (right). (*N* = 3). **C** RT-qPCR relative quantification of GPX4 mRNA 48 h post-transfection with control or ZEB1-targeting siRNA. Error bars represent +/- mean error (*n* = 4). (*N* = 2). **D** Cytotoxicity curve and corresponding IC50 values for SUM159PT cells treated with 0-200 nM RSL-3 for 72 h. Measured using the Promega CellTiter Glo viability assay. IC50 values were calculated using linear Nonlinear regression analysis. (*N* = 3). Unless otherwise indicated, error bars represent the standard deviation of the mean.

Our data clearly show that sensitivity to ferroptosis is a prominent phenotype in the SUM159PT anastatic cells. To further investigate the mechanism regulating this sensitivity to ferroptosis, we evaluated expression levels of ZEB1, an EMT transcription factor previously shown to regulate sensitivity to ferroptosis through its regulation of enzymes involved in PUFA metabolism [23, 25, 26]. Western blot analysis showed a significant increase in ZEB1 expression in the SUM159PT and MDA-MB468 anastatic cells (Fig. 4A and Supplementary Fig. 5A), as well as increased expression of the EMT transcription factor Twist in the SUM159PT cells (Supplementary Fig. 6A) when compared to parental cells. Since SUM159PT cells are already partially mesenchymal, they express higher levels of vimentin and lower levels of E-cadherin (Supplementary Fig. 6A). This is a potential reason as to why the SUM159PT GFP+ cells do not express changes in these markers, as the cells are exhibiting a partial EMT program [27]. Furthermore, these cells also express higher levels of Cancer Stem Cell marker CD44 (Supplementary Fig. 6B). To further probe the potential link between ZEB1 and increased sensitivity to ferroptosis, we determined whether suppressing the expression of ZEB1 would reverse the sensitivity of the SUM159PT anastatic cells to ferroptosis. As shown in Fig. 4B–D, not only did the ZEB1 knockdown rescue SUM159PT anastatic cells from their increased sensitivity to ferroptosis, but it also resulted in a reversal of GPX4 protein expression, which was increased, demonstrating that ZEB1 regulates GPX4 expression and sensitivity to ferroptosis in these anastatic cells. It has previously been shown that ZEB1 suppresses GPX4 expression at the transcriptional level [26]. To determine if ZEB1 was regulating GPX4 transcriptionally in the GFP + SUM159PT anastatic cells, we performed RT-qPCR to measure the relative mRNA quantity of GPX4 during ZEB1 knockdown. These results confirmed the down-regulation of GPX4 by ZEB1 in our model, indicated by the increase in mRNA levels during ZEB1 knockdown (Fig. 4C). These data are consistent with previous reports that ZEB1 directly suppresses GPX4 expression [26].

**Fig. 5:**
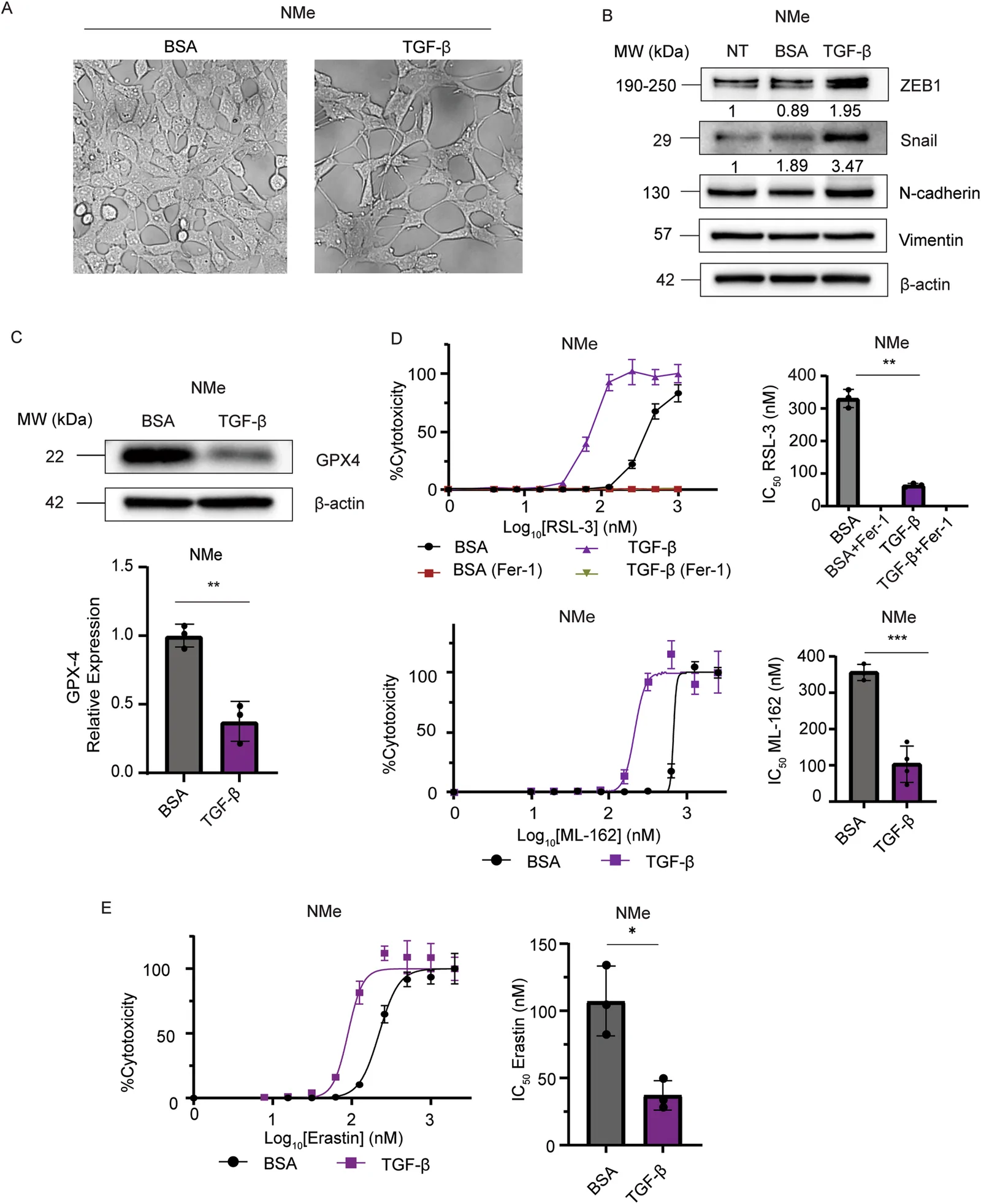
EMT stimulation of NMe cells increases sensitivity to ferroptosis and induces alterations to ferroptosis-associated metabolites. **A** Phase-contrast images of NMe cells after 6-day treatment with TGF- β. Taken using a Zeiss Colibri light microscope. **B** Western blot showing the expression of proteins involved in EMT in NMe cells treated with TGF- β for 6 days. β-actin serves as the loading control. (*N* = 3). **C** Western blot showing the expression of GPX4 in NMe cells treated with TGF-β for 6 days. Quantified via densitometric analysis relative to the β-actin loading control. Significance was determined via an unpaired t-test assuming a Gaussian distribution. (*N* = 3). **D** Cytotoxicity curve and IC50 values for NMe cells treated with 0-200 nM RSL-3 and ferrostatin-1 (top) and 0-2.5μM ML-162 (bottom) for 72 h. Cells were pre-treated with TGF-β for 72 h prior to co-treatment with the inhibitor and TGF-β in a 96-well plate for an additional 72 h. Cell death was quantified using Sytox green stain via Incucyte imaging and normalized using Invitrogen HCS CellMask Stain. IC50 values were calculated via non-linear regression, and outliers were identified using Grubbs’ Test. *N* = 3 (top); *N* = 4 (bottom). **E** Cytotoxicity curve and corresponding IC50 values for NMe cells treated with 0-20 μM Erastin. Cells were treated, and percent cytotoxicity was determined as described in (**D**). (*N* = 3). Unless otherwise indicated, error bars represent the standard deviation of the mean.

To determine whether this phenotype extends beyond SUM159PT cells, we employed a second model involving TGF-β–induced EMT in normal mouse mammary epithelial (NMe) cells. NMe cells were subjected to a 6-day treatment of Transforming Growth Factor–β (TGF-β) of 5 ng/uL to stimulate EMT-associated expression of ZEB1. This resulted in morphologic changes associated with EMT and up-regulation of ZEB1, Snail, and Twist EMT markers (Fig. 5A, B). Mirroring what was shown in the SUM159PT GFP+ cells, EMT stimulation of NMe cells resulted in a sharp down-regulation of GPX4 expression that corresponded to an increased sensitivity to ferroptosis when treated with RSL-3, ML-162, or Erastin (Fig. 5C–E).

Because ZEB1 is known to regulate enzymes involved in lipid metabolism, we wanted to determine if loss of ZEB1 reduced lipid peroxide levels in the SUM159PT anastatic cells [25]. Following siRNA-mediated ZEB1 knockdown, the BODIPY 665/676 probe was used to measure lipid peroxidation. Loss of ZEB1 resulted in decreased lipid peroxidation (Supplementary Fig. 6C), further supporting the role of ZEB1 in sensitizing the SUM159PT anastatic cells to ferroptosis.

### ZEB1-mediated acquired sensitivity to ferroptosis is required for survival of breast cancer cells from chemotherapy-induced apoptosis (anastasis)

**Fig. 6:**
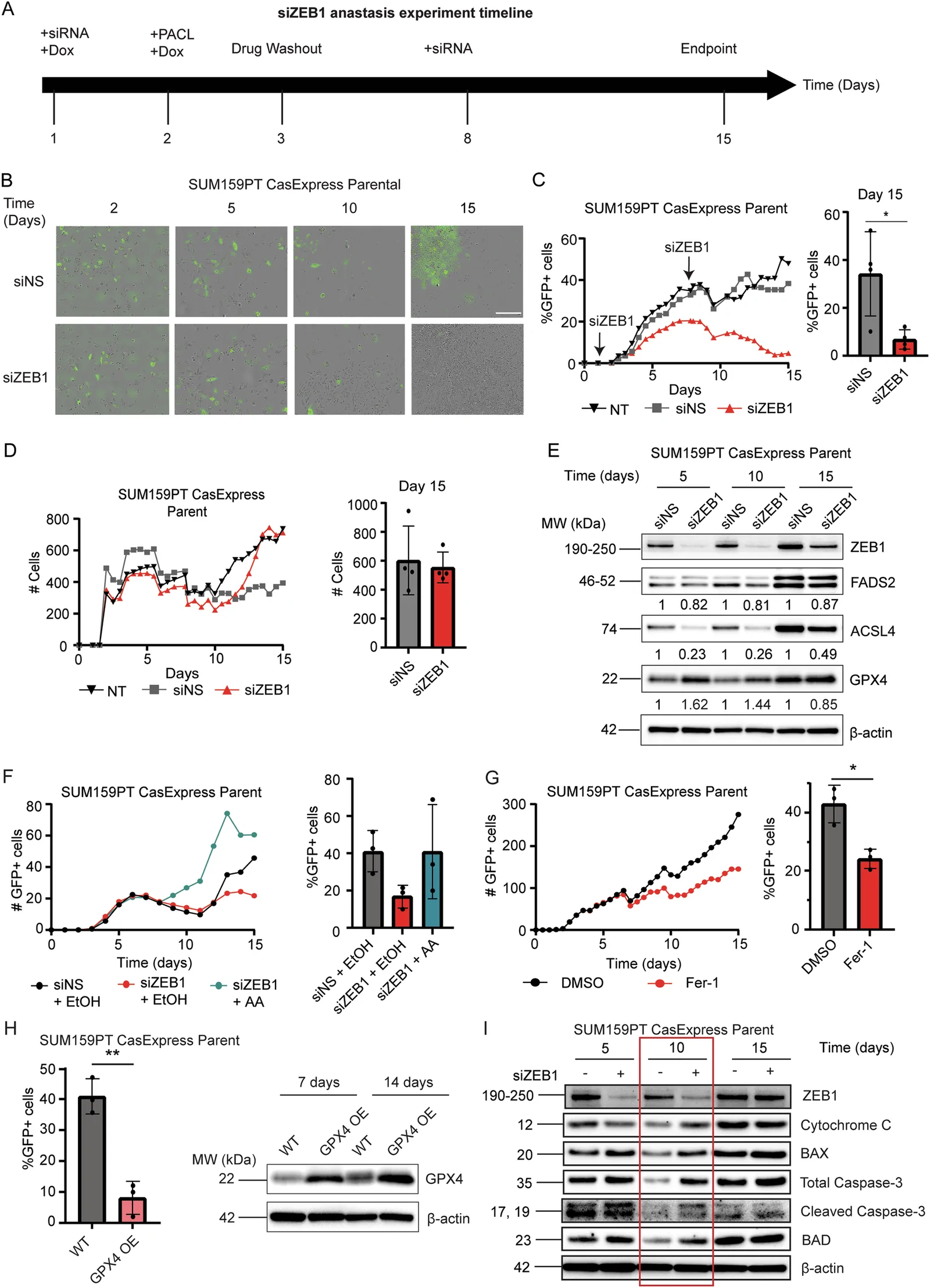
ZEB1 and its regulation of ferroptosis sensitivity are required for SUM159PT cells to undergo anastasis. **A** Schematic representing the experimental timeline. Cells were treated with siRNA on day 0 and day 8 to ensure maintenance of the ZEB1 knockdown. **B** Incucyte time-lapse imaging of control and siZEB1 knockdown SUM159PT CasExpress cells after treatment with 20 nM paclitaxel and Doxycycline. Scale bar in top right corner indicates 300 µm. (*N* = 4). **C** Quantification of the percentage of GFP+ cells relative to the total number of cells. Images were captured, and both GFP+ and cell-by-cell counts were quantified by Incucyte software every 12 h over the course of 15 days. (*N* = 4). **D** Quantification of the total number of cells for each treatment over the 15-day period. (*N* = 4). **E** Western blot showing expression of ZEB1 and ferroptosis enzymes under ZEB1 regulation for each treatment at the indicated time points. Β-actin acts as the loading control. (*N* = 2). Fold changes are indicated below the relevant band and were quantified relative to SUM159PT Parent cells via densitometry analysis in Bio-Rad Image Lab. **F** Left: Number of GFP+ cells quantified by Incucyte software every 24 h over the course of 15 days. Cells were cultured as in (**A**), with the addition of 10 μM arachidonic acid from day 2 onwards. Right: Quantification of the percentage of GFP+ cells was calculated relative to the total number of cells on day 15. GFP+ and total cell counts were quantified by Incucyte software. (*N* = 3). **G** Left: Number of GFP+ cells quantified by Incucyte software every 24 h over the course of 15 days. Right: Quantification of the percentage of GFP+ cells was performed as in (**C**). Cells were cultured as in Fig. 1B with the addition of 2uM Ferrostatin-1 following removal of paclitaxel and continued for the duration of the experiment. Left: Quantification of the percentage of GFP+ cells 14 days following paclitaxel treatment, as described in (**G**). (*N* = 3). **H** Quantification of the percentage of GFP+ cells in SUM159PT CasExpress cells with either wild-type or overexpression of GPX4 14 days following paclitaxel treatment, as described in (**G**). Cells were cultured as described in Fig. 1B. (*N* = 3). **I** Western blot showing expression of ZEB1 and anti-apoptotic enzymes under ZEB1 regulation for each treatment at the indicated time points. Β-actin acts as the loading control. (*N* = 2). Unless otherwise indicated, error bars represent the standard deviation of the mean.

The identification of the central role of ZEB1 in the metabolic phenotype of the anastatic GFP+ cells and EMT-stimulated NMe cells led us to determine whether ZEB1 might also play a role in the acquisition of anastatic survival after chemotherapy-induced apoptosis. We treated SUM159PT CasExpress cells with siZEB1 or control siRNA for 24 h to induce knockdown of ZEB1. Cells underwent 2 rounds of siZEB1-mediated knockdown of ZEB1 over the course of the experiment – one treatment 24 h prior to chemotherapy exposure, and 1 treatment at the midway point on day 8 (Fig. 6A, C). 24 h after the first siZEB1-mediated knockdown, SUM159PT CasExpress parental cells were treated with 20 nM paclitaxel for 24 h before drug washout (Fig. 6A). Cells were then subjected to Incucyte time-lapse imaging for a recovery period of 14 days where the number of GFP+ cells was quantified relative to the total number of cells (Fig. 6C, D). Interestingly, ZEB1 knockdown resulted in a significant decrease in the relative number of GFP+ cells on day 15, but not the total number of surviving cells (Fig. 6C, D). We also consistently observed that at approximately day 10, the cells undergo a “crisis” marked by a decrease in viability, after which there is an expansion of the GFP + , anastatic population (Fig. 6B, C). Cells treated with the non-specific control siRNA appear to recover from the paclitaxel-induced crisis after day 10; however, when ZEB1 is knocked down, the cells are no longer capable of recovering, revealing a crucial role for ZEB1 in survival from chemotherapy-induced apoptosis (Fig. 6B, C).

Because ZEB1 upregulates the expression of ferroptosis promoting proteins – fatty acid desaturase 2 (FADS2), which catalyzes the addition of double bonds to long chain PUFAs, and ACSL4, which activates long chain PUFAs for insertion into the membrane during the recovery of SUM159PT anastatic cells from chemotherapy (Fig. 6E)—we next sought to determine whether supplementation with the ferroptosis-promoting PUFA, arachidonic acid, could rescue ZEB1 knockdown-mediated suppression of anastasis (accumulation of GFP+ cells) in response to chemotherapy [25]. Remarkably, supplementation with 10 µM arachidonic acid following removal of paclitaxel rescued the ability of cells to undergo anastasis and survive chemotherapy-induced apoptosis in the absence of ZEB1 (Fig. 6F), demonstrating that the ZEB1-mediated upregulation of PUFAs is essential for recovery of TNBC cells from chemotherapy-induced caspase activation. Concomitant with its up-regulation of ferroptosis-promoting enzymes during recovery from taxol-induced caspase activation, ZEB1 also suppresses the expression of various pro-apoptotic proteins (Fig. 6I). This effect, most notably on the day 10 “crisis point”, combined with its simultaneous up-regulation of ferroptosis-promoting proteins, further emphasizes the critical role of ZEB1 as a master regulator of anastasis.

Due to this involvement of PUFAs in the survival of TNBC cells from chemotherapy-induced apoptosis and the increased sensitivity of the SUM159PT cells to ferroptosis, we next wanted to determine if sensitivity to ferroptosis itself is required for survival from chemotherapy-induced apoptosis (anastasis). Again, we exposed SUM159PT and Hs578t CasExpress cells to paclitaxel and quantified the number of GFP+ cells during a 2-week recovery period. Following removal of paclitaxel, cells were treated with Fer-1, an inhibitor of ferroptosis that scavenges lipid peroxides, for the duration of the recovery period. As shown in Fig. 6G and Supplementary Fig. 7, inhibition of lipid peroxidation by Fer-1 significantly reduced the percentage of anastatic GFP+ cells, demonstrating a requirement for ferroptosis sensitivity in the acquisition of anastasis. To further support this novel finding, we overexpressed GPX4 in the SUM159PT CasExpress cells, treated them with the same transient dose of chemotherapy, and observed the accumulation of GFP+ cells after 14 days. Again, this resulted in a significant decrease in the number of anastatic GFP+ cells (Fig. 6H), further supporting evidence that ferroptosis sensitivity is required for survival from chemotherapy-induced apoptosis.

Collectively, these data suggest that ferroptosis sensitivity, regulated by ZEB1, is essential for the survival of TNBC cells from chemotherapy-induced apoptosis.

### Anastatic GFP+ cells accumulate in vivo as a result of chemotherapy treatment

**Fig. 7:**
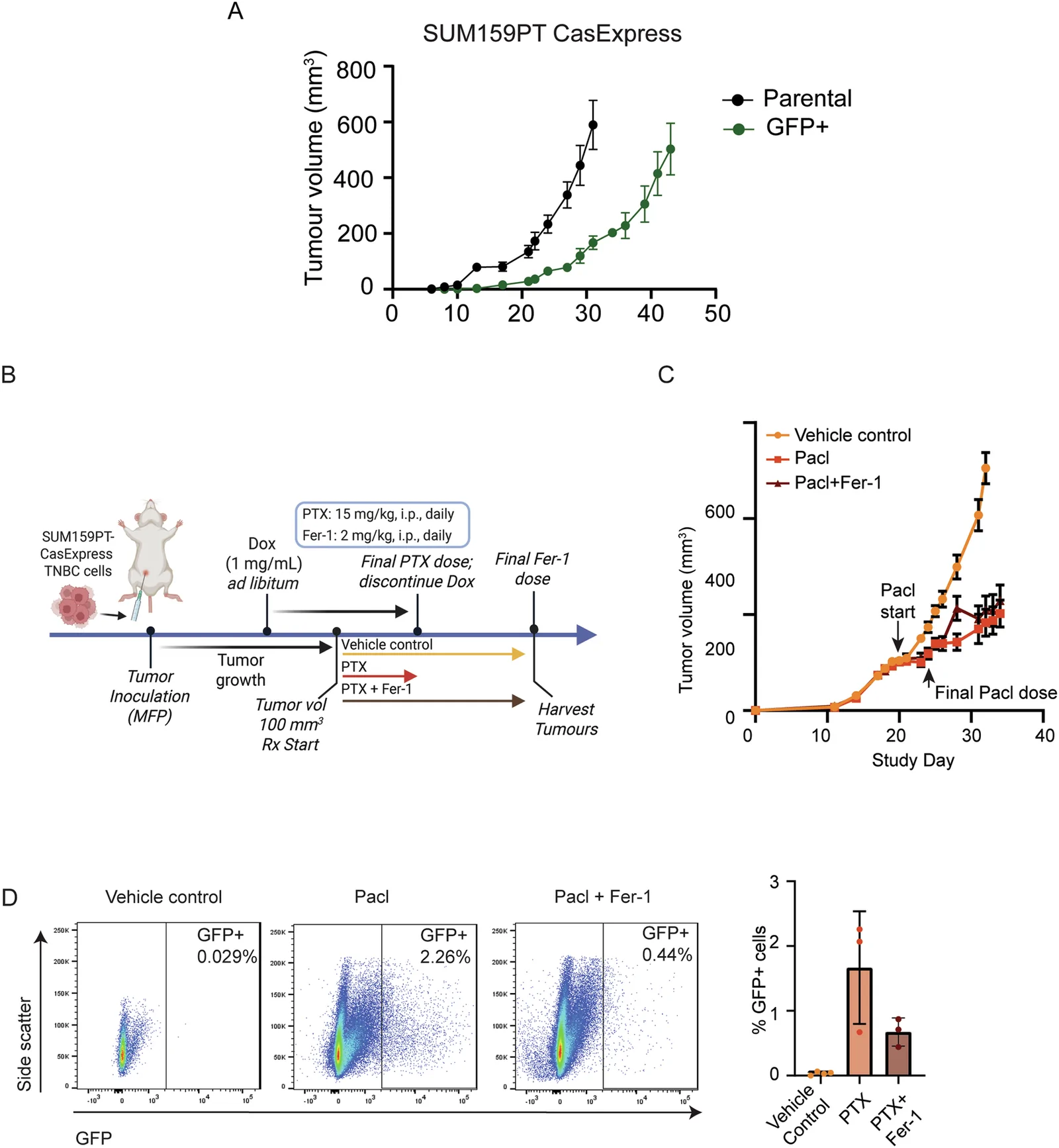
Accumulation of GFP+ cells in vivo. **A** Graph showing tumor volume of each TNBC cell line. Cells were inoculated in female NSG mice in the mammary fat pad (*n* = 4). **B** Schematic of study design. **C** Tumor growth curves of SUM159PT CasExpress tumors inoculated in female NSG mice in the mammary fat pad. Tumors treated as indicated. (*n* = 8) **D** FACS-based sorting of GFP+ cells from tumors in (**C**). (*n* = 3).

Having established that TNBC cells undergo anastasis as a resistance mechanism in response to chemotherapy in vitro, we next sought to determine whether anastasis contributes to tumor recurrence in vivo. To begin, we validated the ability of the SUM159PT GFP+ cell line to form tumors in vivo following injection into the mammary fat pad (MFP) of female NSG mice (Fig. 7A). This demonstrated that tumor cells surviving chemotherapy induced by apoptosis are indeed capable of forming tumors, confirming their potential relevance in tumor recurrence. Next, we determined whether anastasis also constitutes a resistance mechanism in vivo by treating mice harboring parental SUM159PT CasExpress tumors with chemotherapy to trigger tumor regression, and then evaluating recurrent tumors for the presence of GFP+ cells. As depicted in Fig. 7B, SUM159PT CasExpress tumors were established orthotopically in the MFP of NRG mice and monitored for tumor growth until tumors reached ~100mm^3^. Subsequently, mice were treated with paclitaxel (15 mg/kg, three daily i.p. doses) to suppress tumor growth, either alone or in combination with Fer-1 (2 mg/kg, daily i.p.) (Fig. 7C). Mice were supplied with doxycycline-containing water prior to paclitaxel treatment to trigger expression of the CasExpress system. Recurrent tumors were harvested at endpoint and assessed for the presence of GFP+ cells using FACS (Fig. 7D). Remarkably, not only did we observe the accumulation of GFP+ cells in the recurrent tumors, but we also observed an approximately 2-fold *decrease* in GFP+ cell accumulation in mice treated with Fer-1 (Fig. 7D). These data validate our in vitro findings in an in vivo context, and further reinforce the major role that ferroptosis sensitivity plays in cells that survive chemotherapy-induced apoptosis.

### ZEB1 expression is associated with chemotherapy resistance in anastatic cells in vivo and in patient-derived xenografts

Our data clearly show an important role for ZEB1-regulated ferroptosis sensitivity in the survival of cells following chemotherapy-induced apoptosis in vitro. Thus, we next sought to investigate if this mechanism is relevant in vivo. Established tumors were harvested and IHC analyses confirmed increased expression of ZEB1, localized to the nuclei, in the anastatic GFP+ tumors (Supplementary Fig. 8A). Consistent with our in vitro siZEB1 knockdown data, which showed that ZEB1 suppresses GPX4 and promotes expression of ACSL4, IHC analyses showed the GFP+ tumors also exhibit decreased GPX4 and increased ACSL4 expression compared to parental tumors (Supplementary Fig. 8A), thus validating the ZEB1-mediated regulation of ferroptosis sensitivity in vivo.

After determining that the ZEB1 regulation of ferroptosis is present in vivo in our paclitaxel-resistant SUM159PT tumors, we next wanted to assess if ZEB1 could be used as a biomarker to evaluate resistance to common TNBC chemotherapy agents paclitaxel and cisplatin in patient-derived xenograft (PDX) models. To accomplish this, we utilized TNBC patient-derived xenografts which had been rendered resistant to either cisplatin or paclitaxel [28]. Mice were treated with 3-4 rounds of an appropriate regimen of either paclitaxel or cisplatin for resistant tumors, and 1 round of either chemotherapy agent for sensitive tumors. IHC evaluation of these tumors revealed increased ZEB1 expression in both paclitaxel- and cisplatin-resistant tumors, consistent with a role for ZEB1 as a critical effector in promoting resistance of TNBC cells to chemotherapy-induced apoptosis (Supplementary Fig. 8B).

**Fig. 8:**
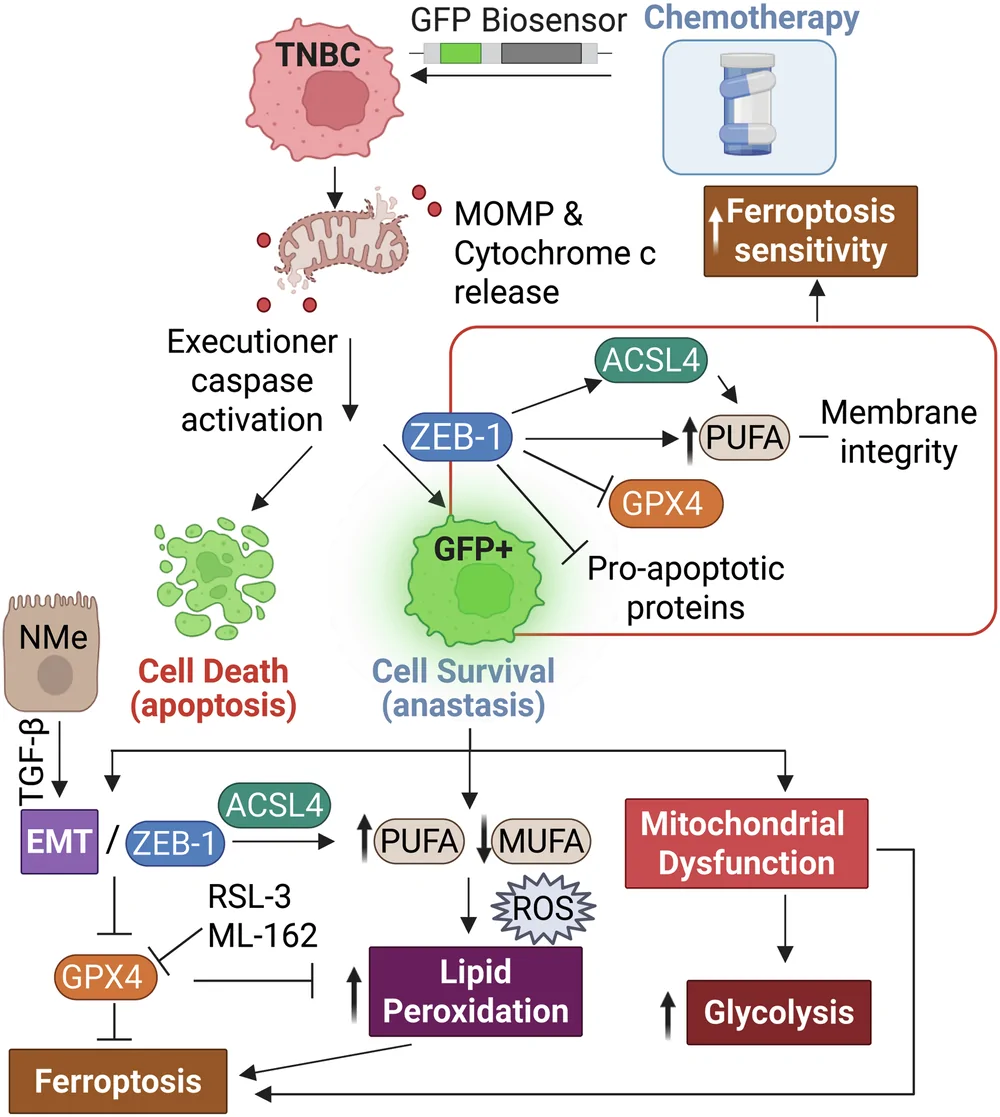
Schematic representing the molecular and metabolic rewiring in SUM159PT anastatic cells. Schematic highlights the molecular and metabolic rewiring mechanisms identified in the SUM159PT anastatic cells in this study. “Pro-apoptotic proteins” represents Cytochrome C, BAX, total caspase-3, cleaved caspase-3, and BAD.

Collectively, these data identify ZEB1 as a master regulator in the acquisition of resistance to apoptotic cell death by sensitizing cells to ferroptotic cell death (Fig. 8). This mechanism is present in vivo, where increased ZEB1 expression is associated with GPX4 suppression and increased ACSL4 – both hallmarks of increased sensitivity to ferroptosis. ZEB1 is then present in preclinical TNBC PDX models, whereby tumors resistant to paclitaxel and cisplatin exhibit increased nuclear expression of ZEB1.

## Discussion

The discovery of novel therapeutics is essential to improve outcomes in patients experiencing recurrent tumors, which remain a major barrier to effective and complete cancer treatment. There is a growing body of work that demonstrates the ability of cancer cells to survive transient chemotherapy-induced executioner caspase activation, a phenomenon which was previously thought to destine the cell for apoptotic death. The survival of cells that experience transient executioner caspase activation has been termed anastasis [6, 9]. Despite the progress that has been made towards understanding features of cells surviving chemotherapeutic treatment, there is a lack of work describing the mechanisms and markers to detect tumors that may be capable of resisting chemotherapy treatment through the process of anastasis, whether these cells represent DTP cells, and if these cells undergo molecular and metabolic rewiring processes. Here, by using the CasExpress reporter system, we isolated a population of TNBC cells surviving chemotherapy-mediated executioner caspase activation to explore and identify molecular and metabolic vulnerabilities in these cells for novel therapeutic targets.

Our primary findings revealed that tumor cells surviving paclitaxel-induced executioner caspase activation (GFP + ) exhibit a more stem-like invasive phenotype and are indeed more resistant to paclitaxel treatment, suggesting that these cells could contribute to tumor recurrence. Strikingly, the SUM159PT GFP+ anastatic cells demonstrated an increased susceptibility to ferroptosis-mediated cell death, resulting from ZEB1-mediated down-regulation of GPX4 and promotion of PUFA enzymes ACSL4 and FADS2 [25, 26]. Together, these findings demonstrate that TNBC cells capable of surviving chemotherapy-mediated apoptosis may give rise to a population of cells that may be simultaneously more aggressive and metastatic, yet be more susceptible to an alternative form of cell death, ferroptosis, due to metabolic re-wiring. Identification of such cells in therapy-resistant recurrent tumors could provide an opportunity to treat recurring tumors by targeting ZEB1, or treatment with ferroptosis-inducing agents, including inhibitors of GPX4 or SLC7A11 [23, 29, 30].

A further critical finding of our work is the demonstration of the role of ZEB1 as a focal point for the adaptive metabolic rewiring that allows TNBC cells to survive chemotherapy-mediated apoptosis. By knocking down ZEB1 prior to chemotherapy treatment and quantifying the relative amount of GFP+ cells after a 15-day recovery period, we found that ZEB1 knockdown significantly reduced the ability of the SUM159PT cells to undergo anastasis. The ZEB1-expressing cells can escape apoptotic activation of executioner caspases, while ZEB1 knockdown cells cannot, identifying ZEB1 as a critical effector in the acquisition of anastasis. Specifically, this was found to be due to ZEB1-mediated promotion of PUFA expression and a concomitant increase in lipid peroxidation, exemplified by an increase in GFP+ cell accumulation during supplementation with arachidonic acid and a decrease in GFP+ cell accumulation during treatment with the antioxidant Fer-1, demonstrating that sensitivity to ferroptosis is required for anastasis. Although this is an interesting mechanistic observation, further pre-clinical validation is required to determine if ferroptosis inducers can prevent tumor recurrence. Not only do these data identify ferroptosis as a targetable vulnerability to prevent recurrence of TNBC tumors, but also reveal a previously unrealized link between ferroptosis and apoptosis by identifying a role for ferroptosis-promoting PUFAs and lipid peroxides in survival from executioner caspase activation. This paradigm is also present in vivo, whereby the SUM159PT GFP+ tumors exhibit increased nuclear expression of ZEB1 and a corresponding increase in ACSL4 and decrease in GPX4 expression. Remarkably, TNBC PDX models rendered resistant to paclitaxel and cisplatin chemotherapy regimens also exhibit a significant increase in ZEB1 expression. Not only does this identify ZEB1 as a master regulator of molecular and metabolic rewiring in the acquisition of anastasis, but it also demonstrates that ZEB1 may serve as a marker for tumors that have survived chemotherapy treatment by anastasis. A limitation here is that, due to heterogeneous spatial intra-tumoral expression of GPX4, we are unable to demonstrate decreases in GPX4 expression in the PDX models. Spatial transcriptomics or proteomics may resolve the expression further, and this will need to be validated if ZEB1 and GPX4 are to be used as biomarkers.

While we have shown this intriguing phenotype whereby EMT induces a sensitivity to ferroptosis that is actually required for the survival of TNBC cells from chemotherapy-induced apoptosis, there is likely heterogeneity present in the mechanisms used by TNBC to undergo anastasis. Our findings set the stage for further studies to explore alternate mechanisms of resistance to chemotherapy-induced apoptosis in TNBC. Nevertheless, our findings do suggest that high expression of ZEB1 and low expression of GPX4 may serve as biomarkers to identify patients whose tumors are prone to metabolic rewiring that enhances ferroptosis sensitivity and anastasis-mediated survival after chemotherapy, and who may benefit from ferroptosis-targeting therapies.

## Materials and methods

### Cell lines and cell culture

The SUM159PT (RRID: CVCL_5423) cell line was a gift from S. Gorski and was maintained in DMEM containing 10% FBS, 1X nonessential amino acids, 5ug/mL insulin, and 1ug/mL hydrocortisone. The NMe cell line was cultured as described previously and incubated with 5 ng/uL TGF-β or vehicle (4 mM HCl, 1 mg/mL bovine serum albumin) for the indicated amount of time [31]. The MDA-MB-468 cell line (ATCC) was maintained in DMEM containing 10% FBS. The Hs578t cell line was a gift from S. Aparicio and was maintained in DMEM containing 10% FBS and 10ug/mL insulin. The HCC1806 cell line was a gift from S. Aparicio and was grown in RPMI + 10% FBS. All cells were grown at 37 °C in a humidified atmosphere containing 5% carbon dioxide (CO_2_). All cell lines have been recently STR validated and mycoplasma tested by a commercial testing facility.

### Generation of SUM159PT cells stably expressing the CasExpress biosensor

HEK293T cells were plated in 6-well plates at 5 × 10^4^ cells/cm^2^ in DMEM + 10% FBS. The following day, cells in each well were transfected with 1 μg each of pCW57-Lyn111-NES-DEVD-FLPO-Hygro and pCDH-FRT-STOP-FRT-ZsGreen plasmids, together with 0.9 μg CMV-dr8.91 packaging plasmid and 0.1 μg VSV-G/pMD2.G envelope plasmid in OPTI-MEM media containing TransIT-LT1 (Mirus Bio: Madison, Wisconsin, USA) according to the manufacturer’s instructions and incubated overnight [6]. The following morning, media was removed and 1 mL/well of OPTI-MEM was added. Media was removed 48 h later and centrifuged at 2000 rpm for 5 min to pellet any cellular debris. Supernatants containing lentivirus were filtered through a 0.45 μm filter and added to SUM159PT human breast cancer cells at an MOI of 0.3 for 24 h. The following day, the virus-containing media was removed, and cells were subcultured in DMEM + 10% FBS. Transduced cells were selected using puromycin, followed by selection with hygromycin.

### Generation of CasExpress GFP+ anastatic cell lines

SUM159PT cells stably expressing the CasExpress biosensor were cultured overnight in 10 cm plates to 60–80% confluence. Following the incubation, cells were treated with 20 nM paclitaxel or vehicle in the presence of 1 μg/ml doxycycline (dox) for 24 h. The following day, cells were washed twice with 1X PBS to remove the chemotherapy and cultured in media containing 1 μg/ml dox for 72 h. Cells were then washed once with 1X PBS and subcultured for 2–3 weeks to allow for recovery and return to proliferation. To enrich for GFP-positive post-anastasis cells, 1 × 10^7^ cells were suspended in FACS buffer (1X PBS, pH 7.4 + 3% FBS), washed twice in cold PBS, and resuspended in FACS buffer. Cells were subsequently incubated with propidium iodide (1:10 000) (Sigma-Aldrich: Saint Louis, Missouri, USA) to label live/dead cells. Sorting was performed on a FACSAria III (BD Biosciences: Mississauga, Ontario, Canada). Cells were sorted into a solution consisting of 1:1 media and FBS. Cells treated with dox and vehicle were used to establish gating parameters for collection of GFP-positive cells. All samples were analyzed using FlowJo v10 (FlowJo LLC: Ashland, Oregon, USA). Sorted GFP-positive, “anastatic” cells were subcultured and expanded for downstream analyses.

### siRNA gene knockdowns

Cells were cultured overnight in 6-well plates to 50% confluence. The following day, cells were transfected with siLentFect (Bio-Rad Laboratories: Hercules, California, USA) following the manufacturer’s protocol. Cells were then treated with 20 nM of ZEB1-targeting siRNA (Qiagen SI04951072; Germantown, Maryland, USA) in Opti-MEM for 4 h, after which media containing 20% FBS was added and cells were incubated for an additional 20 h. Cells were then washed with 1X PBS, collected, and plated for the indicated downstream experiment.

### Sphere limiting dilution assay

Cells were harvested in Hank’s Buffered Salt Solution (HBSS) + 2% FBS, centrifuged at 1000 rpm for 5 min and resuspended in HBSS. This was performed twice to remove all traces of trypsin and FBS. Cells were filtered to obtain a single-cell dilution, and the Sphere Limiting Dilution Assay was carried out as previously described [32].

For quantification of invasive protrusions, images of the largest spheroids from the SLDA (512 cells seeded) were taken on day 7 using the 4x objective in the Incucyte. The number of invasive protrusions was quantified using the Incucyte spheroid invasion analysis software.

### Cell preparation and harvest for LC-MS/MS metabolomics analysis

SUM159PT cells stably expressing the CasExpress biosensor and SUM159PT anastatic (GFP-positive) cells were cultured on 15 cm plates for 72 h to 50–70% confluence. NMe cells were cultured on 10 cm plates in the presence of TGF-β or vehicle for 48 h before being resuspended and replated onto a new 10 cm plate under the same conditions for 48 h. At this point cells were collected and plated onto 15 cm plates under the same conditions for 48 h to 50–70% confluence. Following incubation, cells were washed 3 times in cold 1X PBS while on ice. Cells were then harvested in ice-cold 80% HPLC-grade methanol on dry ice by scraping.

### Metabolomics analysis

LC-MRS/MS of organic acids was performed by the University of Victoria Proteomics Centre. Samples were submitted in 0.8-1.5 mL 80% HPLC-grade methanol. Measurement of metabolites was then carried out by the University of Victoria Proteomics Centre as described below.

Samples were normalized to 1.5 mL in 80% methanol and sonicated at 4 °C for 2 min before being centrifuged at 21,000 × *g*, 5 °C for 10 min. The supernatant was collected for targeted metabolite analysis, and the pellet was used for a BCA protein assay. Analysis of the organic acids used a chemical derivatization method as described by the University of Victoria Proteomics Centre and a serially diluted standard solution of the targeted metabolites [33, 34]. 20uL of each sample was mixed with 80 mM 3-nitrophenylhydrazne and 60 mM EDC-6% pyridine solutions and incubated at 45 °C for 40 min after which 100uL of premade internal standard solution was added. For thiol compounds, a standard solution containing authentic substances of targeted thiol compounds was prepared and serially diluted in 80% methanol to be used as calibration solutions. 20uL of each sample and calibration sample was mixed with 20uL internal standard solution containing isotope-labeled cysteine, glutamic acid, GSH, and GSSG, and 60uL of N-ethylmaleimide solution. For NAD + , NADH, NADP + , and NADPH, a standard solution containing authentic substances of all 4 metabolites was prepared and serially diluted in 80% methanol to be used as calibration solutions. 20uL of each sample and calibration sample was mixed with 80uL internal standard solution containing isotope-labeled NAD+ and NADH. 10uL of each sample for each preparation was injected into a C18 UPLC column and run using UPLC-MRM/MS (Santa Clara, California, USA). Organic acid and thiol assays were run using the 1290 UHPLC system. Organic acid compounds were run while coupled to an Agilent 6495B QQQ mass spectrometer operated in negative-ion detection mode. 0.01% formic acid in water and acetonitrile-isopropanol (1:1) were used as the mobile phase for gradient elution under optimized conditions of LC separation and MS detection. Thiol compounds were run while coupled to an Agilent 6495 C QQQ mass spectrometer operated in positive-ion detection mode. An HFBA buffer and methanol were used as the mobile phase for gradient elution under optimized conditions of LC separation and MS detection. NAD + , NADH, NADP + , and NADPH were run on a UPLC-MRM/MS on a Waters Acquity UPLC system coupled to a Siex QTRAP 6500 Plus mass spectrometer in negative-ion detection mode. Tributylamine buffer and methanol were used as the mobile phase for gradient elution. The calibration solution was used to construct standard curves to produce linear regression curves for the metabolites within each sample.

### Western blotting

Cells were cultured to 50-70% confluency and lysed in RIPA buffer (50 mmol/L Tris-HCl pH 7.6, 150 mmol/L NaCl, 0.1% (w/v) SDS) containing 1 mmol/L Na_3_VO_4_, 2 mmol/L NaF, and cOmplete protease inhibitor. Protein concentration was determined using a BCA assay, and 5 μg of protein was run on a 1–4% gradient Bis-Tris gel in 1X NuPAGE MOPS SDS running buffer at 120-150 V. Gels were transferred onto PVDF membranes for 1 h in cold Tris-glycine transfer buffer with 15% methanol at 100 V. Membranes were fixed in 100% methanol for 2 min then incubated with primary antibody in 5% BSA in TBST overnight at 4 °C. The following day, membranes were washed 3 times with TBST for 15 min at room temperature, then incubated with HRP-conjugated secondary antibody in 5% milk in TBST for 1 h. Membranes were washed 3 times with TBST for 15 min then visualized using Supersignal West Femto chemiluminescence reagent and the Bio-Rad ChemiDoc XRS+ imaging system. Raw blots can be found in the “Raw Blots” file.

### qPCR

Cells were cultured overnight in 6-well plates to 60-70% confluence. The following day, cells were harvested and pelleted by spinning at 1000 × *g* for 5 min. The pellet was resuspended in 1x PBS and centrifuged at 1000 × *g* for 5 min to remove all traces of media. RNA extraction was performed using the Invitrogen (Carlsbad, California, USA) PureLink RNA Mini Kit (12183018 A) following the manufacturer’s protocol, and cDNA was produced using the Bio-Rad Laboratories iScript cDNA synthesis kit (#1708890). The qPCR reaction was carried out following the Bio-Rad Prime PCR recommended protocol.

### PCR

Indicated CasExpress cell lines were cultured overnight to 80% confluency at 37 °C, 5% CO_2_. The following day, cells were treated with 1ug/mL doxycycline for 72 h after which 20 nM paclitaxel was added, and cells were incubated in the presence of doxycycline for an additional 24 h. The following day, media was removed, and cells were washed with 1X PBS, trypsinized, and pelleted. DNA extraction was performed using the ThermoFisher (Waltham, Massachusetts, USA) Invitrogen PureLink Genomic DNA Mini Kit (K18200) following the manufacturer’s protocol. PCR was performed using the Bio-Rad T100 Thermal Cycler (#1861096) targeting the CasExpress zsGreen plasmid using primers as previously described [11]. Samples were run on a 0.8% agarose gel in 1X Tris-acetate EDTA buffer at 100 V for 30-40 min.

### Cytotoxicity assays

CasExpress cells were seeded at 1200 cells in a 96-well plate and incubated overnight at 37 °C, 5% CO_2_. The following day, cells were treated with the indicated serial dilution of drug in media containing 1X Sytox Orange (ThermoFisher Scientific, S34861) (CasExpress cell lines) or Sytox Green (S7020) (NMe cell line). Cells were then incubated with the drug for either 72 or 96 h and then scanned using the appropriate channel in the Incucyte. Cells were normalized using the Cell-by-Cell function (CasExpress cell lines) or using HCS stain (ThermoFisher Scientific, H32712) (NMe cell line). Cytotoxicity curves were generated using GraphPad Prism by transforming X values using X=log (X) and then normalized to the first mean (0%) and last mean (100%). Cytotoxicity curves containing Ferrostatin-1 treatment were normalized to 100% using the highest values in samples not treated with Ferrostatin-1. IC_50_ values were calculated using nonlinear regression. Outliers were identified using Grubbs’ method.

### Clonogenic assay

Indicated cell lines were seeded at 2000 cells per well in 6-well plates and incubated for 7 days until colonies formed. Cells were fixed in 1 mL of ice-cold 100% methanol and fixed at –20 °C for 10 min. Methanol was removed, and plates were allowed to dry at room temperature ( ~ 30 min). 0.5% crystal violet in 20% ethanol was added to each well and incubated on a shaker for 30 min. Crystal violet was washed off in water until the water was clear. Plates were allowed to air-dry and were stored in a dark place until imaging using the Bio-Rad ChemiDoc XRS+ imaging system using the Coomassie blue channel.

### Seahorse assays

SUM159PT CasExpress cell lines were seeded at 10,000 cells in a 96-well plate. After seeding, cells were allowed to adhere at room temperature on a flat surface before being incubated overnight at 37 °C, 5% CO_2_. The following day, cells were subjected to either the Seahorse XFe Mito Stress Test (#103015-100) or Glycolytic Rate Assay (#103344-100) using the Seahorse Agilent XFe analyzer (Agilent). Indicated parameters were quantified and normalized by fluorescent microscopy (1:1000 Hoechst stain) using the Wave software (Agilent).

### Flow cytometry

Cells were seeded at 25 000 cells in a 6-well plate and followed the experimental outline in Fig. 6A. For analysis, cells were washed with 1X HBSS and incubated with 2 µM BODIPY 665/676 (Thermo Fisher) at 37 °C, 5% CO_2_ for 15 min. Cells were washed with 1X HBSS and trypsinized for 10 min 37 °C, 5% CO_2_. Cells were collected in HBSS + 2% FBS (HF) and centrifuged for 5 min at 1000x rpm to pellet cells. Cells were then resuspended in 500-1000 µL HF and incubated on ice for 30 min before analyzing on BD LSRII Fortessa (BD Biosciences). Data were analyzed in FlowJo v10.8.1 (BD Biosciences). For CellROX (Thermo Fisher Scientific, #C10422) staining, cells were cultured and analyzed as previously described [35].

### Animal studies

All experimental animal procedures were carried out at the BC Cancer Animal Resource Centre (ARC) in accordance with protocol A22-0103 approved by the institutional Animal Care Committee (ACC) at the University of British Columbia, Vancouver. The studies are compliant with all relevant ethical regulations regarding animal research. All animals were housed within the Animal Resource Centre (ARC) at the BC Cancer Research Institute in Vancouver, Canada. All mice were housed in ventilated cages in a pathogen-free, environment-controlled room at 19–21 °C. The relative humidity ranged between 40% and 70%, and a photoperiod of 12 h light and 12 h darkness was provided. Food and water were provided ad libitum. Environmental enrichment included nesting material, hiding places such as huts, and standard bedding.

For tumor growth studies, SUM159PT CasExpress+ parental and GFP+ cells (1.0 x 10^6^ cells/animal, suspended in 100 μL 80% matrigel/20% sterile saline) were injected into the mammary fat pad of naïve11-12 week old female NOD.Cg-*Prkdc*^*scid*^ *Il2rg*^*tm1Wjl*^/SzJ (NSG) mice. A total of 8 mice entered the study. Animals were housed in groups of 4 mice per cage, and cages were grouped into 2 arms (parental cells, *n* = 4; GFP+ cells, *n* = 4). Animals harbouring the same cell line were housed together and animals were not randomized. Each cell line was harvested independently and injected sequentially on the same day to ensure the integrity of each cell line. Tumors were measured 3 times per week using digital calipers to monitor tumor growth, and tumor volumes were calculated using the modified ellipsoid formula (l × w2 × π/6). Inclusion and exclusion criteria based on digital caliper measurements were not established a priori, and all animals were included in the analysis. For each experimental group, the primary experimental endpoint was defined as the day on which the average tumor volume exceeded 500 mm^3^. At endpoint, the tumors were harvested, fixed in formalin, and embedded in paraffin for downstream immunohistochemical analyses. The number of animals per group was chosen based on data from previous studies [35, 36]. The investigators were not blinded to the identity of the study groups and were therefore aware of group allocation at all stages of the experiment. While not formally registered, protocols and schedules were prepared prior to the initiation of the study.

Body weights were recorded three times per week during the course of the study as a measure of animal health. A clinical observation and health monitoring record was also maintained for each individual animal throughout the study, and animals were monitored 3-5 times/week. Steps were taken to reduce animal distress during the studies, including provision of sunflower seeds to maintain animal weight. The humane endpoints for this study were based on a balance between measurable signs such as weight loss (greater than 20% loss in weight), food and water intake, as well as reduced levels of activity, behavioral changes, and physical appearance (e.g., coat and eye appearance). No adverse events were reported during the study period, and all animals involved in the study reached the experimental endpoint prior to the humane endpoints.

For tumor regression studies, SUM159PT-CasExpress cells (1.0 × 10^6^ cells/animal, suspended in 100 μL 80% Matrigel/20% sterile saline) were injected into the MFP of 24 naïve 8–9-week-old female *NOD.Cg-Rag1tm1Mom Il2rgtm1Wjl/SzJ* (NRG) mice, and animals were housed in groups of 3 to 4 mice per cage throughout the study. Tumor growth was monitored using measurements with a digital caliper, and volumes were calculated using the modified ellipsoid formula (l × w2 × π/6). All mice were administered doxycycline (dox) (1 mg/mL) in drinking water containing 1% sucrose *ad libitum* starting three days prior to administration of paclitaxel (Pacl) and continuing until the termination of administration of Pacl. Control animals were similarly administered drinking water containing 1% sucrose. When the average tumor volume across the entire cohort of mice reached ~100 mm^3^, mice were mixed to create 3 groups (*n* = 8/group) with similar average tumor volumes in each group, including vehicle control (saline + 5% DMSO), Pacl alone, and Pacl + Fer-1, and were thus not randomized.

Paclitaxel (6 mg/mL stock, DIN 02391465, Accord Healthcare Inc, Kirkland, QC) was diluted to 1.5 mg/mL in saline and administered to mice by intraperitoneal (i.p.) injection at a dose of 15 mg/kg once daily for 3 days. Animals were administered 2 mg/kg of Ferrostatin-1 (Fer-1, HY-100579, MedChemExpress) formulated in 5% DMSO/95% sterile saline once daily by i.p. injection, a dose and frequency that inhibits ferroptosis in vivo [37, 38]. For animals receiving Pacl and Fer-1, Fer-1 was formulated together with Pacl, and animals were administered the combination as a single i.p. injection during the Pacl treatment window. The day following the final dose of Pacl, animals in this group received Fer-1 formulated as described above, and animals received a total of 14 doses. For all mice, administration of dox was terminated the day following the final dose of Pacl, and mice were returned to regular water *ad libitum*.

The administration of both cells and drugs was performed in a staggered fashion by cage to minimize potential confounders between mice in the control and experimental groups. No animals were excluded from analysis.

Body weights were recorded daily during the treatment phase of the study as a measure of animal health. A clinical observation and health monitoring record was also maintained for each individual animal throughout the study, and animals were monitored 2-5 times/week. Steps were taken to reduce animal distress during the studies, including administration of subcutaneous saline injections as needed to mitigate dehydration and provision of sunflower seeds to maintain animal weight. The humane endpoints for this study were based on a balance between measurable signs such as weight loss (sustained loss in weight of greater than 20% from onset of the study), food and water intake, as well as reduced level of activity, behavioral changes, and physical appearance (e.g., coat and eye appearance). No adverse events were reported during the study period, and all animals involved in the study reached the experimental endpoint prior to the humane endpoints. Tumors were harvested for downstream FACS sorting of GFP+ cells.

The investigators were not blinded to the identity of the study groups and were therefore aware of group allocation at all stages of the experiment. The number of animals per group was chosen based on data from previous studies [32, 39]. While not formally registered, protocols and schedules were prepared prior to the initiation of each study.

Immunohistochemical analyses of ZEB1 expression in the chemotherapy-sensitive and resistant PDXs used archived tissues from previously published studies [28].

### FACS analysis of tumors

Tumors were excised, trimmed of residual fur, and washed in 1X PBS to remove excess blood. Tumor dissociation and disaggregation were adapted from a previously established protocol [40]. Tumors were maintained on ice and dissociated into a fine pulp using sterile razor blades. Tumor fragments were then incubated in 3 mL of digestion medium (DMEM + 5% FBS + 1 mg/mL collagenase IV + 0.33 mg/mL DNase) and further dissociated with the plunger of a 3 mL syringe. Samples were incubated at 37 °C for 40 min with gentle agitation.

Following digestion, cell suspensions were filtered through 40 μm cell strainers. Tumor fragments remaining on the strainer were mechanically disrupted using the plunger of a 3 mL syringe, and the strainer was washed with PBS to maximize cell recovery. The resulting single-cell suspension was collected and centrifuged at 1000 rpm for 5 min at 4 °C. Cell pellets were resuspended in HF buffer, washed by centrifugation, and subsequently incubated in 4 mL ACK lysis buffer on ice for 15 min to remove erythrocytes. Cells were washed twice with HF buffer, centrifuging at 1000 rpm for 5 min at 4 °C between washes.

To assess viability, cells were stained with eFluor 780 viability dye (1:1,000 dilution) for 20 min at 4 °C. Following staining, cells were washed with HF buffer and centrifuged at 1200 rpm for 5 min at 4 °C. Cells were then resuspended in PBS containing 1% FBS, filtered through a 40 μm cell strainer, and transferred to pre-labeled FACS tubes. Samples were maintained at 4 °C until FACS was performed on the BD FACS Aria Fusion.

### Immunohistochemistry

Immunohistochemistry was carried out as previously described [36, 41, 42]. Five-micron sections of formalin-fixed, paraffin-embedded tissue were deparaffinized, rehydrated, and antigen retrieval was performed by microwaving in 10 mM citrate buffer, pH 6.0, for 10 min. Sections were incubated with 3% H_2_O_2_ for 15 min to quench endogenous peroxidase activity, followed by blocking for an h at room temperature in blocking buffer (3% skim milk, 1% BSA in PBS). Tissue sections were incubated with primary antibodies diluted in blocking buffer overnight at 4 °C. After 3 washes, the sections were incubated with ImmPRESS HRP antibody for 30 min at room temperature. The DAB staining was carried out using DAB peroxidase HRP Substrate according to the manufacturer’s instructions. Tissues were counterstained with hematoxylin (Leica Biosystems).

### Statistical analysis

Significance of datasets was determined using One-way ANOVA to test for differences in one variable across discrete groups. Where appropriate, Brown-Forsythe and Welch ANOVA tests were applied in the event that equal standard deviations could not be assumed. In the event that only 2 variables were being compared, the parametric unpaired t-test (two-tailed) was used. If standard deviations were not equal, Welch’s correction was used. For both ANOVA and unpaired t-tests, Welch’s correction was applied if the difference between standard deviations was greater than 2-fold. Data are presented as the Mean ± SD. Statistical analyses were performed using Prism v10.1.2 software (GraphPad, La Jolla, CA). For both ANOVA and t-tests, the significance is indicated as follows: *p < 0.05; **p < 0.01; ***p < 0.001; ****p < 0.0001. Exclusion of outlier samples from analysis was determined using Grubbs’ Test. “*n*” denotes technical replicates.

### Ethics statements

All methods were performed in accordance with the relevant regulations. Animal studies were conducted in accordance with protocol A22-0103 approved by the institutional Animal Care Committee (ACC) at the University of British Columbia, Vancouver, Canada.

### Antibodies

The antibodies used in this study are presented in Table 1.

**Table 1:** Antibodies list.

Antibody	Source	Cat #	RRID	Dilution
ZEB1 Rabbit mAb (D80D3)	Cell Signaling Technology	3396	AB_1904164	1:1000
ZEB1	Sigma-Aldrich	HPA027524		1:100
Snail (C15D3) Rabbit mAb	Cell Signaling Technology	3879	AB_2255011	1:1000
Slug (C19G7) Rabbit mAb	Cell Signaling Technology	9585	AB_2239535	1:1000
Vimentin (D21H3) XP Rabbit mAb	Cell Signaling Technology	5741	AB_10695459	1:2000
Mouse Anti-N-Cadherin	BD Biosciences	610920	AB_2077527	1:1000
Anti Vinculin Ab, clone VIIF9 (7F9) Mouse mAb	Millipore Sigma	MAB3574	AB_2304338	1:1000
β-actin Mouse mAb	Sigma-Aldrich	A5441	AB_476744	1:4000
Cleaved caspase-3 (Asp175) (5A1E) Rabbit mAb	Cell Signaling Technology	9664	AB_2070042	1:500
Caspase-3 Rabbit mAb [E83-103]	Abcam	32499	AB_777433	1:1000
ACSL4 (A-5) Mouse mAb	Santa Cruz	sc-271800	AB_10715092	1:1000 (WB) 1:400 (IHC)
SLC1A5 (D7C12) Rabbit mAb	Cell Signaling Technology	8057	AB_10891440	1:1000
CD71 (D7S5Z) Rabbit mAb	Cell Signaling Technology	13208	AB_2798150	1:1000
GLS Rabbit mAb [EP7212]	Abcam	156876	AB_2721038	1:1000
SLC7A11 [EPR8290(2)] Rabbit mAb	Abcam	175186	AB_2722749	1:1000
GCLC (H-5) Mouse mAb	Santa Cruz	sc-390811	AB_2736837	1:1000
GPX4 Rabbit mAb [EPNCIR144]	Abcam	125066	AB_10973901	1:1000 (WB) 1:100 (IHC)
Twist Mouse mAb [Twist2C1a]	Abcam	50887	AB_883294	1:500
E-Cadherin Mouse mAb	BD Biosciences	610181	AB_397580	1:1000
FADS2 Rabbit mAb [EPR27357-82]	Abcam	314317	AB_3665948	1:1000
Cytochrome C [7H8.2C12]	Abcam	13575	AB_300470	1:1000
BAX	Cell Signaling Technology	2772	AB_10695870	1:1000
BAD (11E3)	Cell Signaling Technology	9268	AB_10695002	1:500

Comprehensive list of antibodies used in the study.

Supplementary information is available at Cell Death and Disease’s website.

## Supplementary information

Supplemental figures

Raw blots

## Data Availability

The datasets generated during and/or analysed during the current study are available from the corresponding author on reasonable request.
