# Supplementary material for: Survival of cancer cells following taxol-induced apoptosis is supported by ZEB1-mediated ferroptosis sensitivity
Source: Cell Death Dis. 2026 Sep 2;17(1):912. doi: 10.1038/s41419-026-09242-7 (PMC13714279; doi:10.1038/s41419-026-09242-7)
Supplement: Supplementary file 1 [file 41419_2026_9242_MOESM1_ESM.pdf]

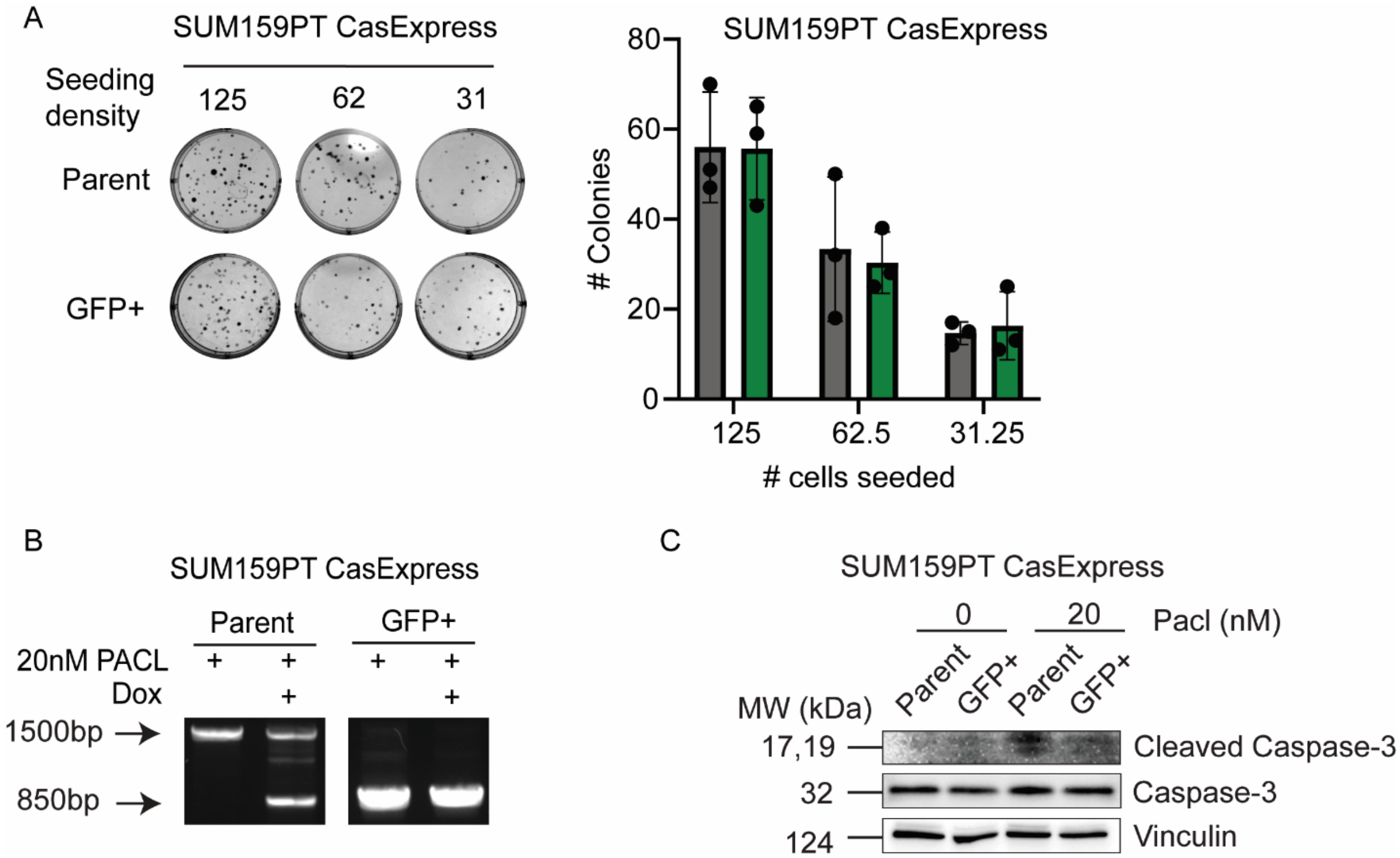


**Supplemental Figure 1. Evaluation of SUM159PT anastatic cells.** (A) Representative images of colonies formed in a clonogenic assay. Number of colonies is quantified in the bar graph on the right (N=3). (B) Agarose gel electrophoresis of the zsGreen construct shown in panel A. Performed on sorted SUM159PT CasExpress cells established from panel B. Expected length of GFP+ phenotype = 864bp. (N=2) (C) Western blot showing expression of cleaved caspase-3. Vinculin serves as the loading control. (N=3). Unless otherwise indicated, error bars represent standard deviation of the mean.

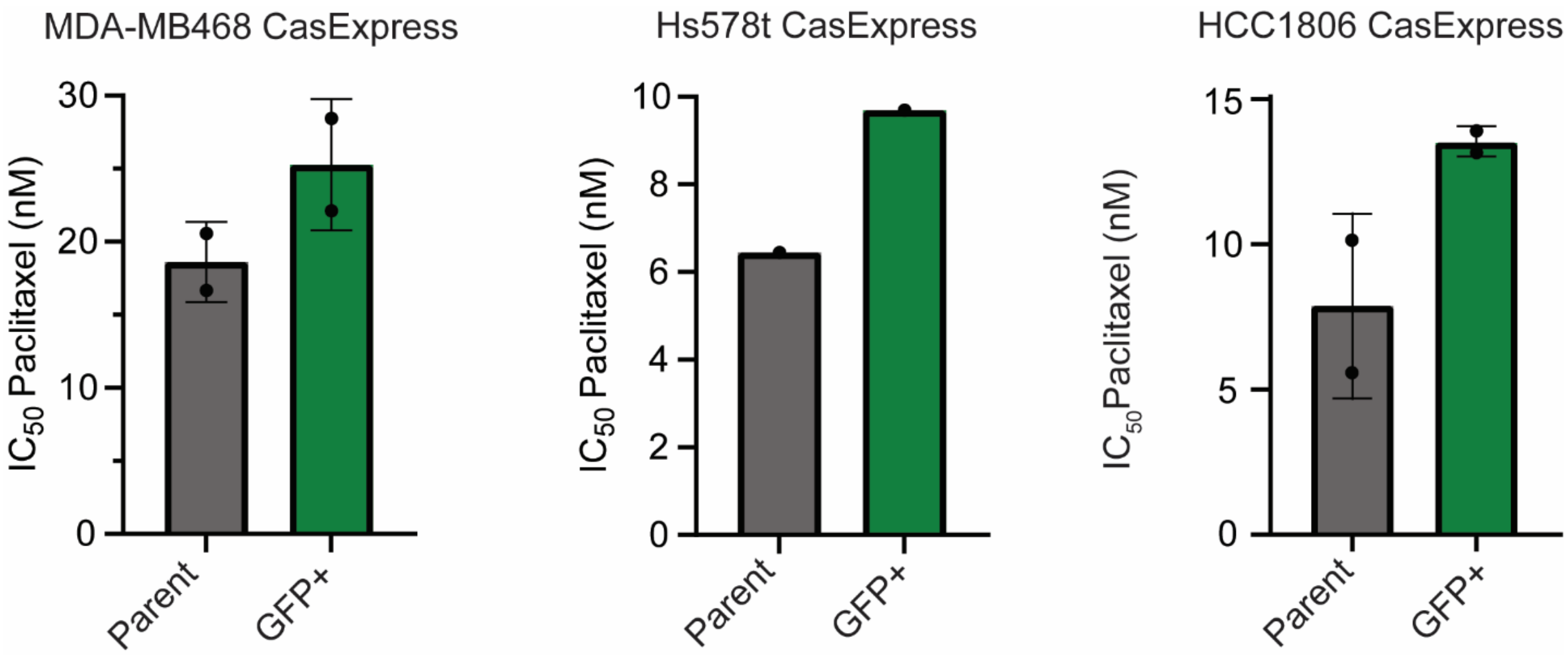


**Supplemental Figure 2. Evaluation of resistance to paclitaxel in anastatic TNBC cell lines.** $IC_{50}$ values for the indicated TNBC cell lines treated with 0-200nM paclitaxel for 24 hours (n=4). Cell death was quantified using Sytox Orange stain via Incucyte imaging and normalized using the Incucyte Cell-by-Cell function. Unless otherwise indicated, error bars represent standard deviation of the mean.

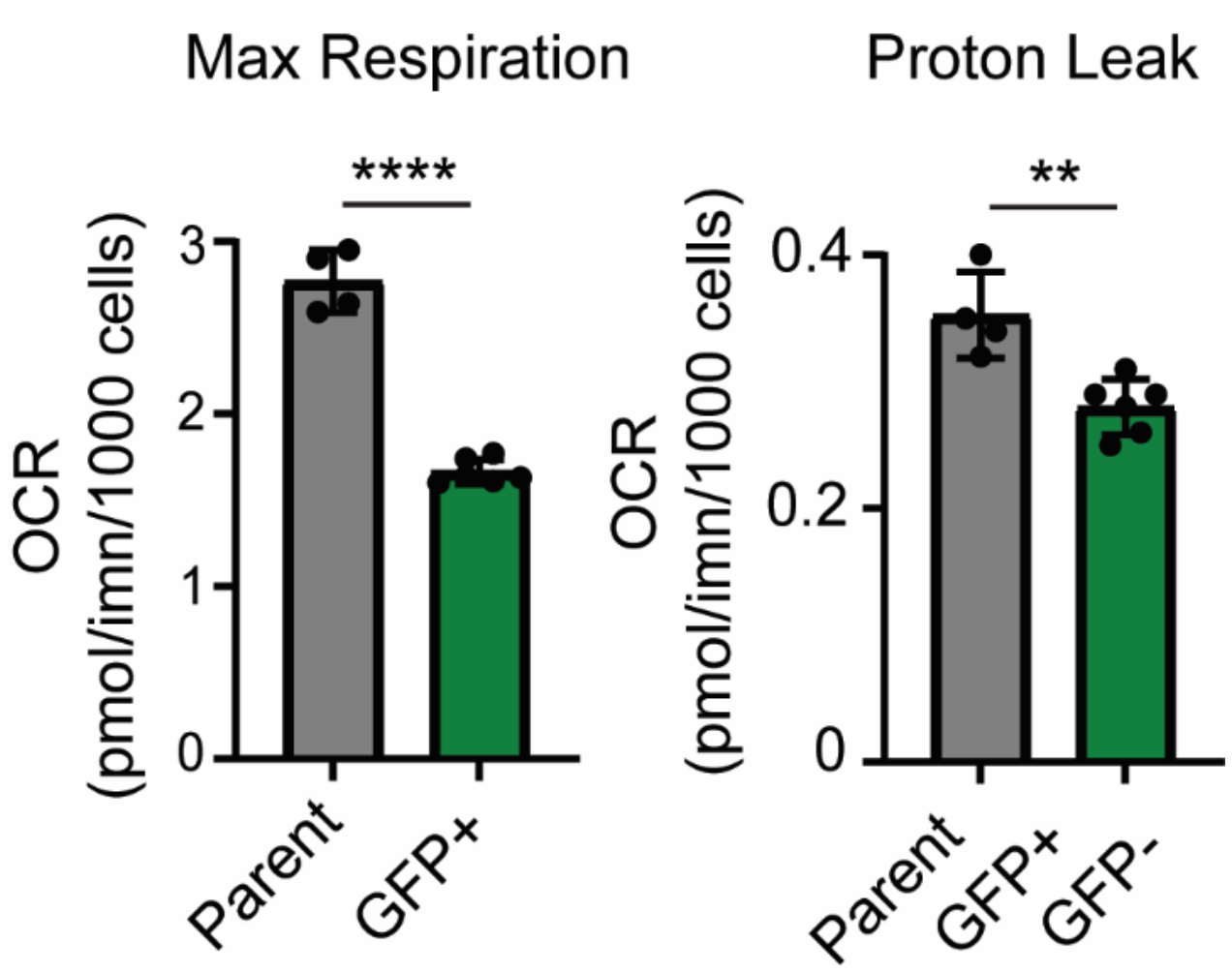


**Supplemental Figure 3. Evaluation of oxidative phosphorylation in SUM159PT CasExpress cells.** Quantification of the indicated parameters determined from the Agilent Seahorse Mito Stress Test. Data was quantified from the oxygen consumption rate using the Agilent Seahorse software. (N=2). Unless otherwise indicated, error bars represent standard deviation of the mean.

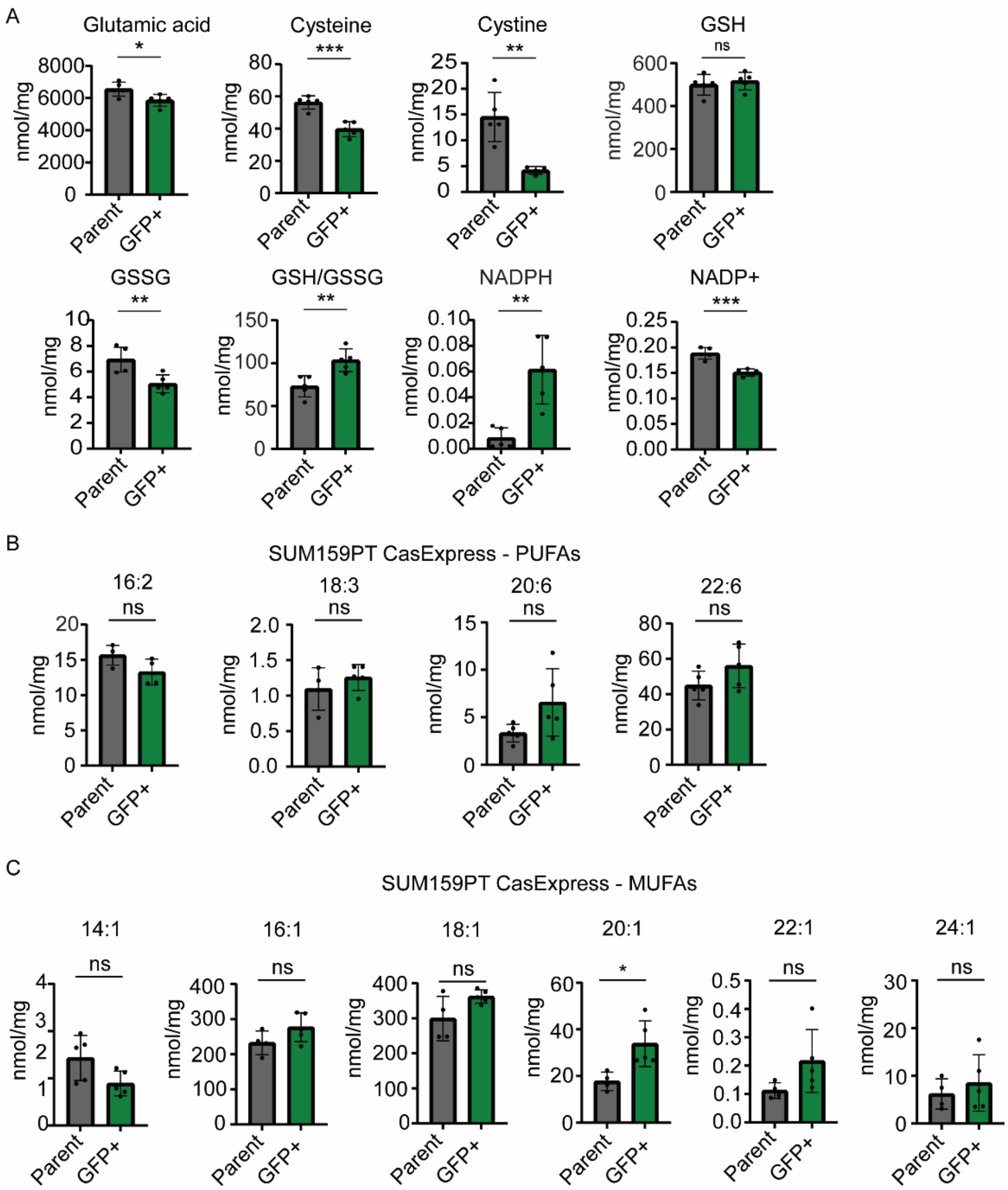

**Supplemental Figure 4. Expression of ferroptosis associated metabolites in SUM159PT parental and GFP+ cells.** (A) Concentrations of SLC7A11-GSH-GPX4 axis metabolites acquired via UPLC-MRM/MS metabolomics analysis. Significance was determined using p-value calculated by the unpaired t-test assuming Gaussian distribution (n=5). (B) Standardized quantification of polyunsaturated fatty acids was performed by UPLC-MRM/MS analysis by the University of Victoria Proteomics and Metabolomic Centre. A BCA assay was used to standardize all samples (n=5). Significance was determined using the p-value calculated by the unpaired t-test assuming Gaussian distribution. Nomenclature represents the total number of carbons in the fatty acid tail to the number of double bonds present. (C) Standardized quantification of MUFAs acquired as in panel B. Unless otherwise indicated, error bars represent standard deviation of the mean.

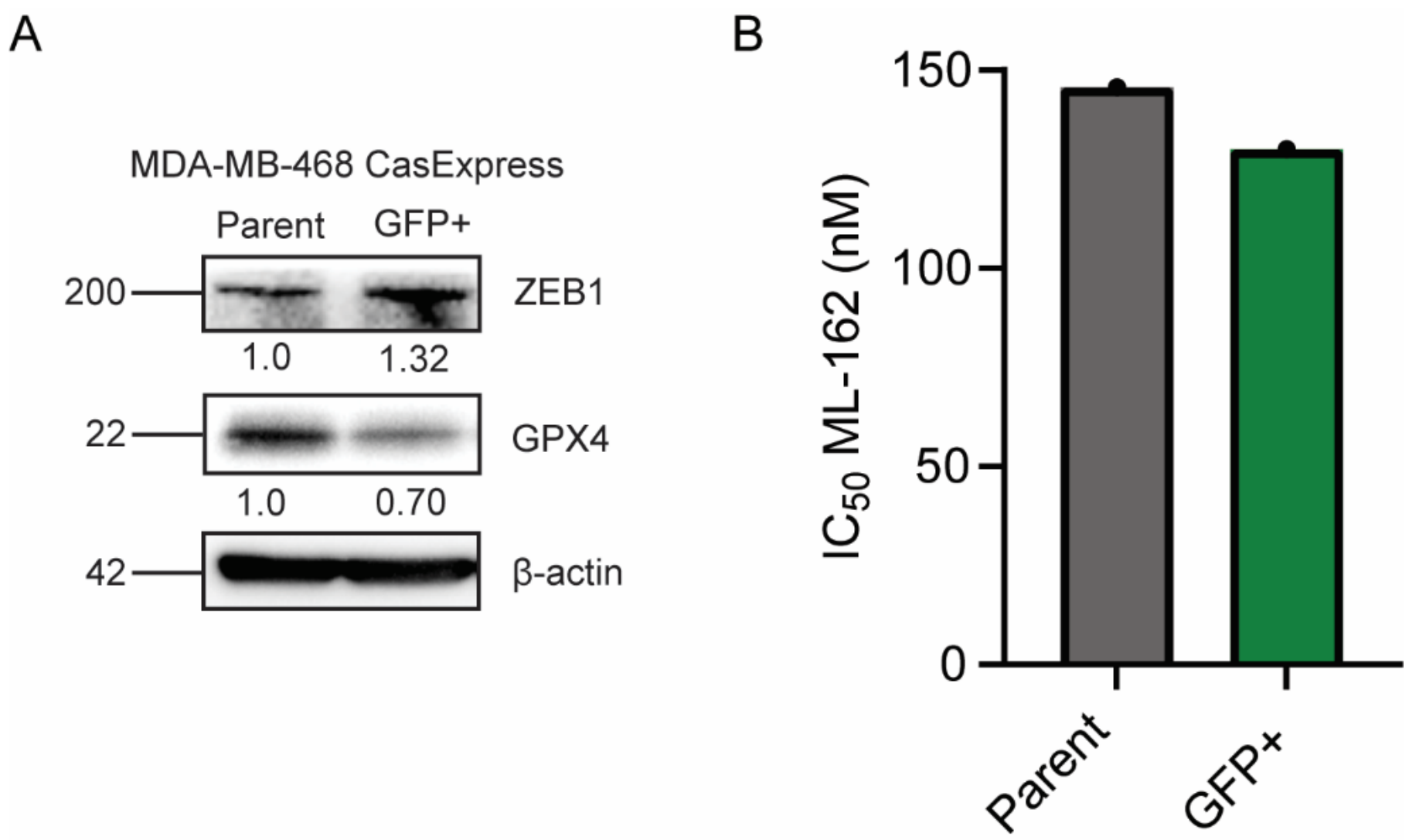


**Supplemental Figure 5. Evaluation of expression of anastasis-associated proteins and ferroptosis sensitivity in MDA-MB468 anastatic cells.** (A) Western blot showing expression of ZEB1 and GPX4. B-actin serves as the loading control. (N=2) (B) IC50 values for SUM159PT cells treated with 0-1µM ML-162 for 72 hours. Cell death was quantified using Sytox Orange stain via Incucyte imaging and normalized using the Incucyte Cell-by-Cell function. IC50 values were derived from the cytotoxicity curve. (N=1). Unless otherwise indicated, error bars represent standard deviation of the mean.

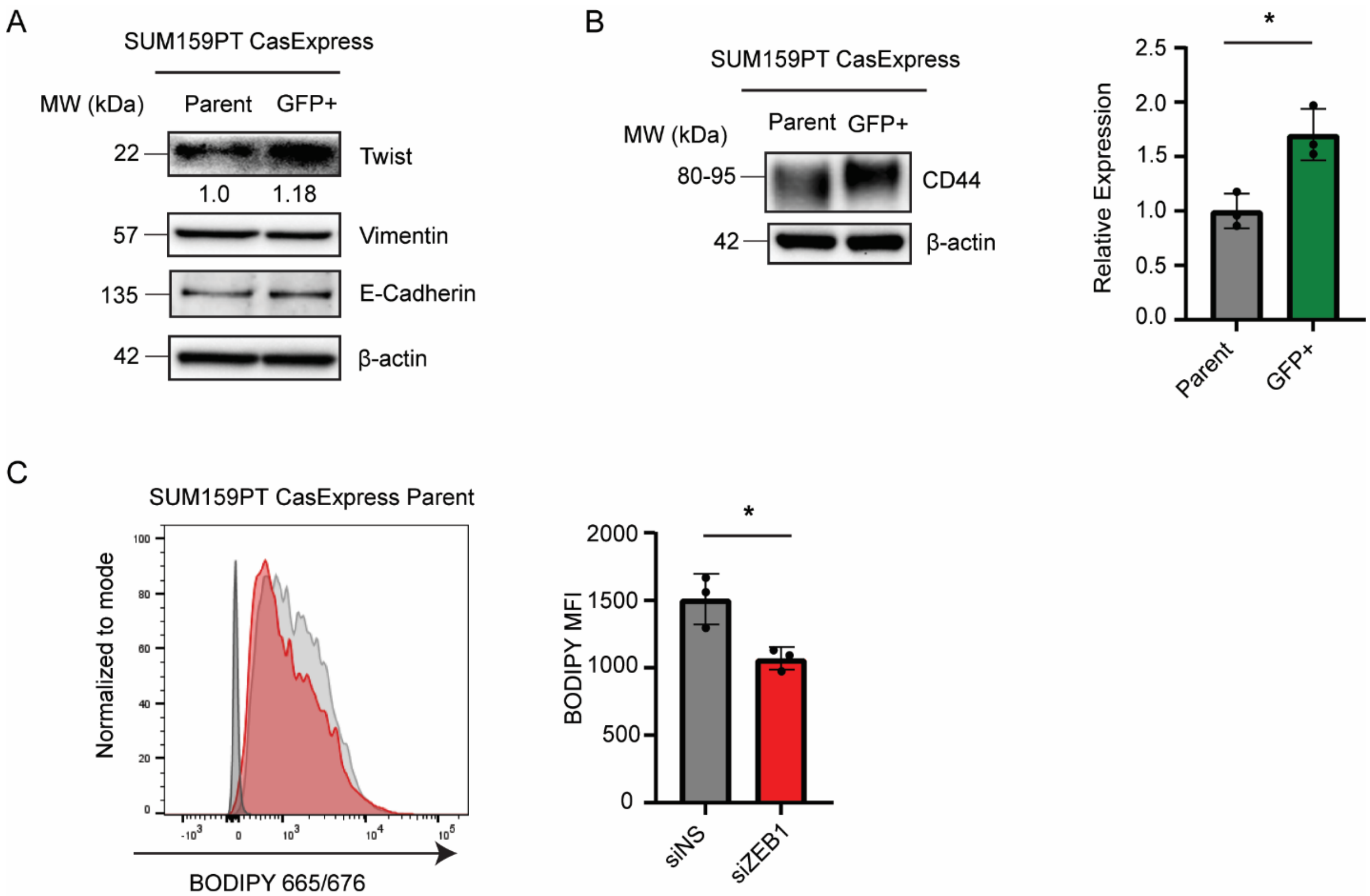


**Supplemental Figure 6. Quantification of EMT associated proteins and lipid peroxidation in SUM159PT CasExpress cells.** (A) Western blot showing expression of EMT effectors. B-actin serves as the loading control. Fold changes are indicated below the relevant band and were quantified relative to SUM159PT Parent cells via densitometry analysis in Bio-Rad Image Lab. (N=3). (B) Western blot showing the expression of cancer stem cell marker CD44 in SUM159PT cells seeded at $5x10^4$ cells per well in a 6-well plate and incubated for 48 hours (left). Quantification via densitometric analysis in Bio-Rad Image Lab relative to the β-actin loading control in SUM159PT cells seeded at $1.5x10^5$ cells per well in a 6-well plate and incubated for 24 hours (right). (N=3) (C) Measurement of lipid ROS 10-days post-treatment with 20nM paclitaxel using BODIPY 665/676 lipid peroxidation probe by flow cytometry. Mean fluorescence intensity quantifed using the geometric mean. (N=3) Unless otherwise indicated, error bars represent standard deviation of the mean.

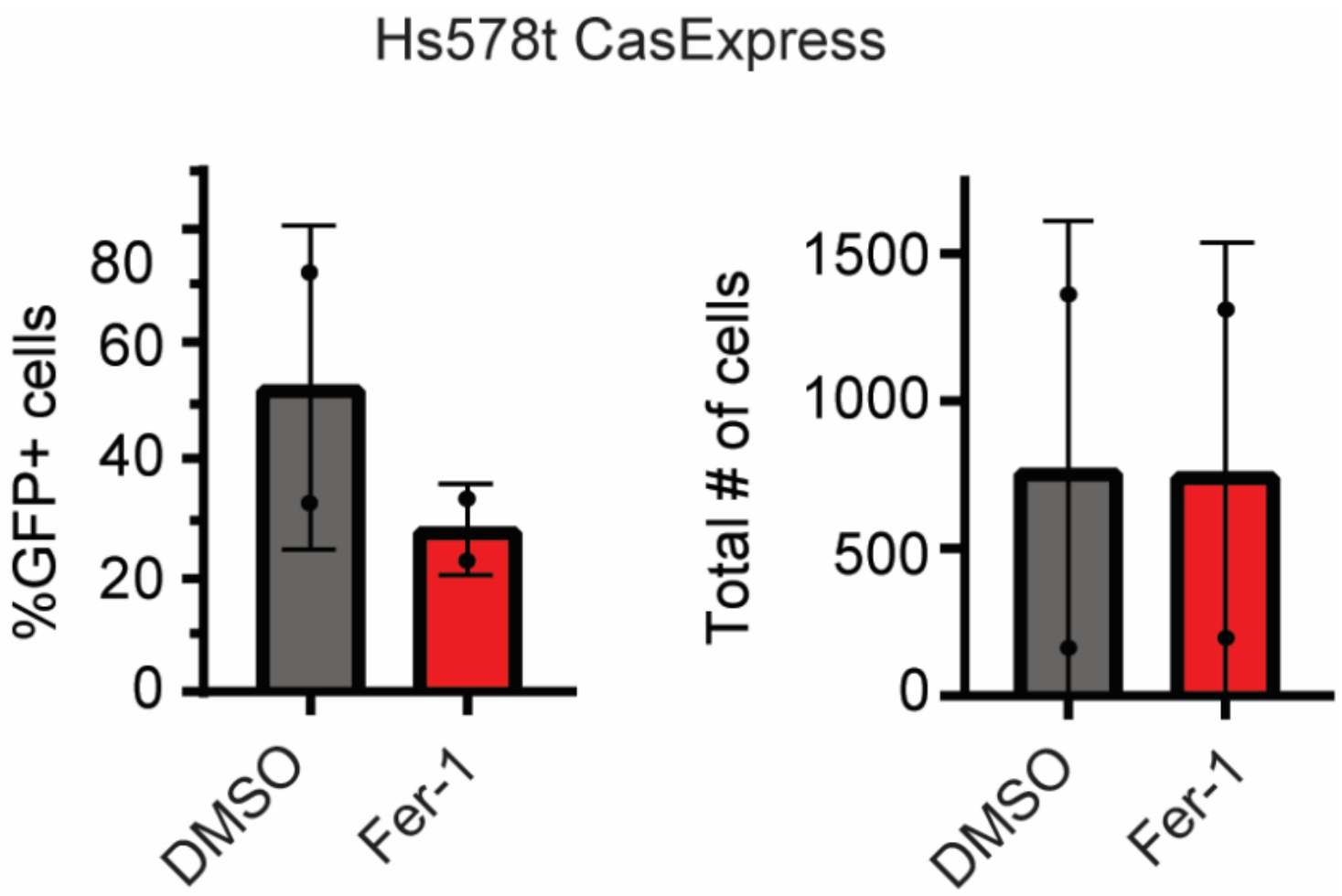


**Supplemental Figure 7. Ferrostatin-1 prevents the accumulation of GFP+ cells following paclitaxel treatment in Hs578t cells.** The quantification of percentage of GFP+ cells is relative to the total number of cells. Images were captured and both GFP+ and cell-by-cell counts were quantified by Incucyte software. Cells were cultured as in Figure 1B with the addition of 2uM Ferrostatin following removal of paclitaxel and continued for the duration of the experiment. (N=2) Unless otherwise indicated, error bars represent standard deviation of the mean.

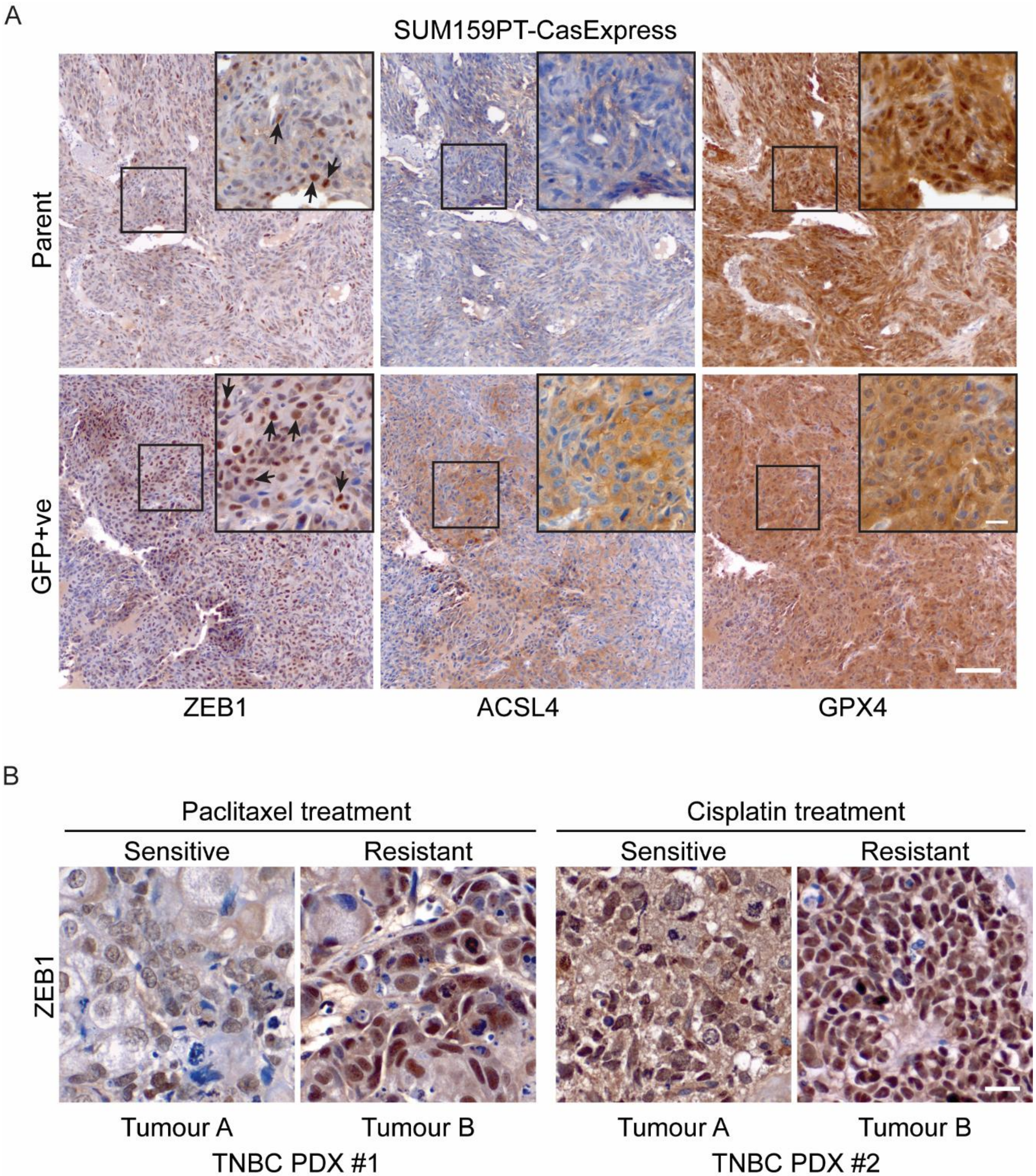


**Supplemental Figure 8. Expression of anastasis-associated proteins in vivo** (A) Images of representative tumour tissue sections from the indicated SUM159PT-CasExpress xenografts stained by immunohistochemistry (IHC; brown signal) for levels of the indicated proteins. Boxes, regions of interest (ROI) shown at higher magnification. Localization of ZEB1 to cell nuclei is indicated (arrows). Scale bars: lower magnification, 100 μm; higher magnification, 20 μm. (B) Images of representative tumour tissue sections from TNBC PDXs treated with the indicated chemotherapy and analyzed by IHC for levels of ZEB1 expression. For each PDX, tumour A is sensitive to chemotherapy and tumour B is resistant to chemotherapy. Scale bar, 20μm.
