# Supplementary material for: Survival of cancer cells following taxol-induced apoptosis is supported by ZEB1-mediated ferroptosis sensitivity
Source: Cell Death Dis. 2026 Sep 2;17(1):912. doi: 10.1038/s41419-026-09242-7 (PMC13714279; doi:10.1038/s41419-026-09242-7)
Supplement: Supplementary file 2 [file 41419_2026_9242_MOESM2_ESM.pdf]

Western blot images in Figure 3

Figure 3C

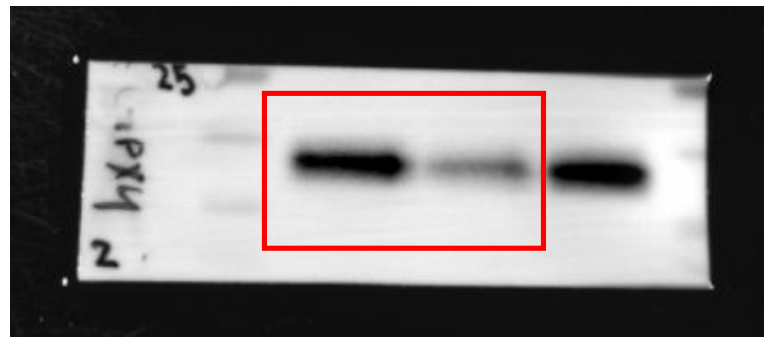

GPX4

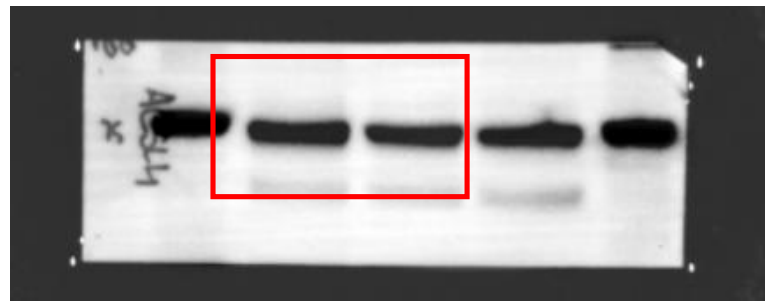

ACSL4

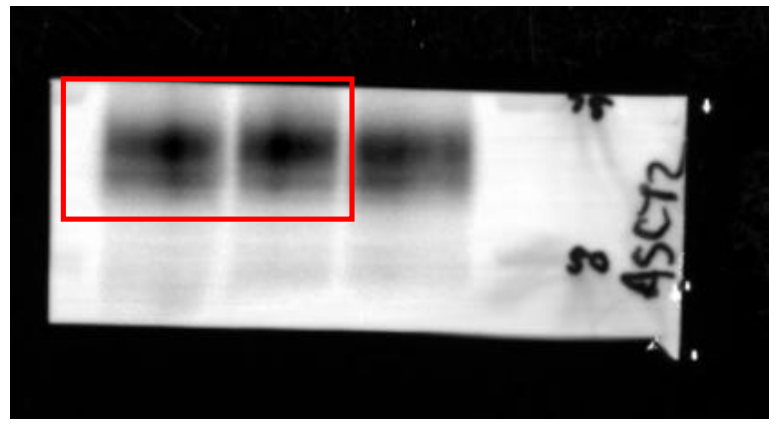

SLC1A5

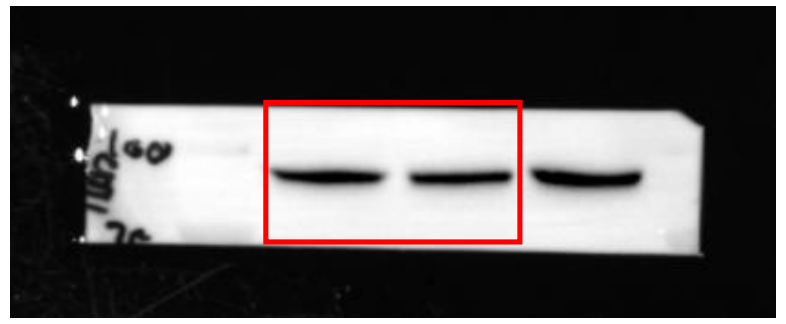

CD71

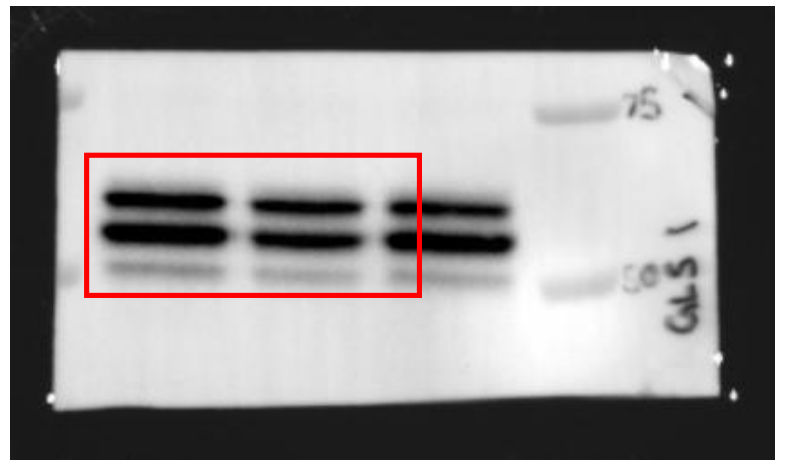

GLS

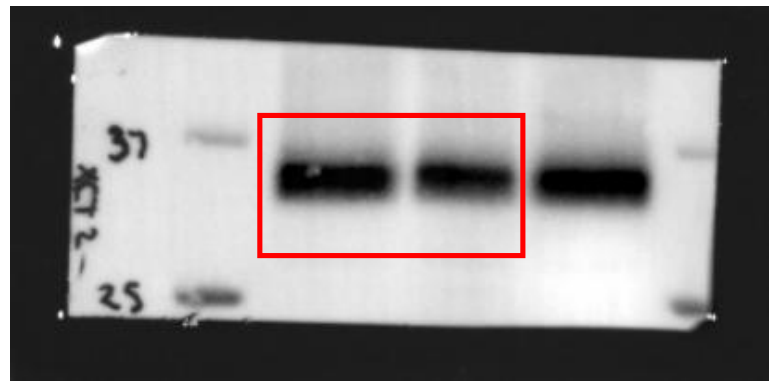

SLC7A11

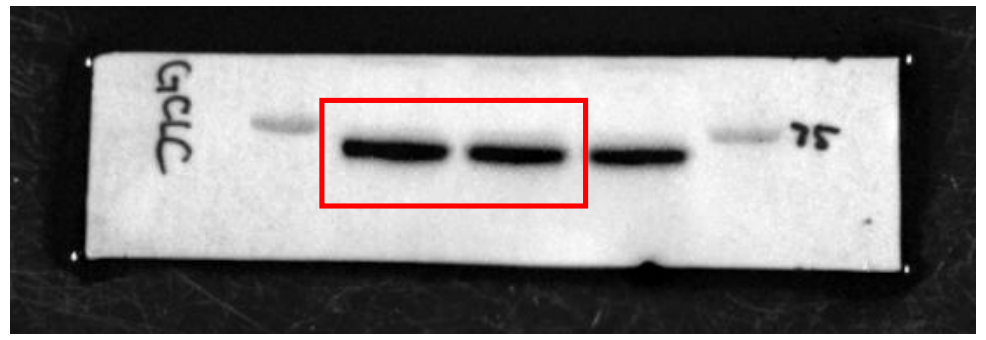

GCLC

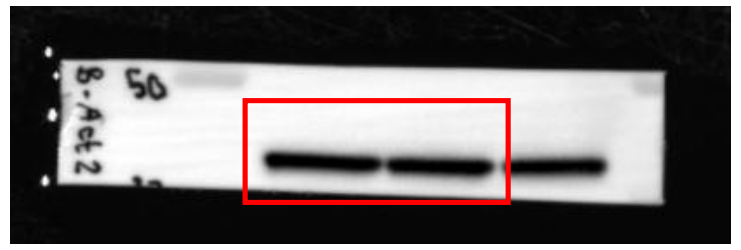

B-actin

Figure 3D

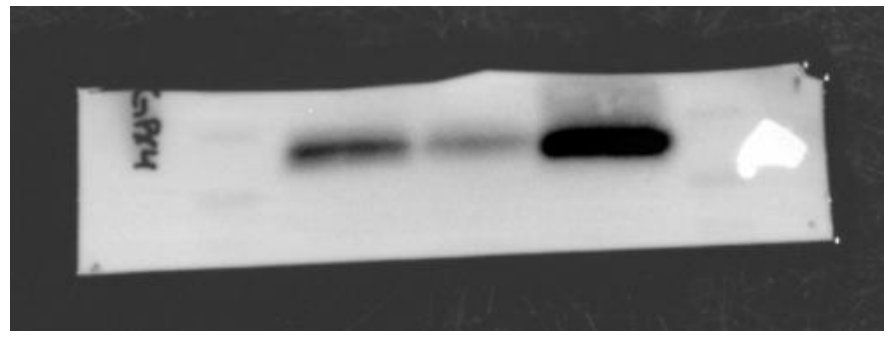

GPX4

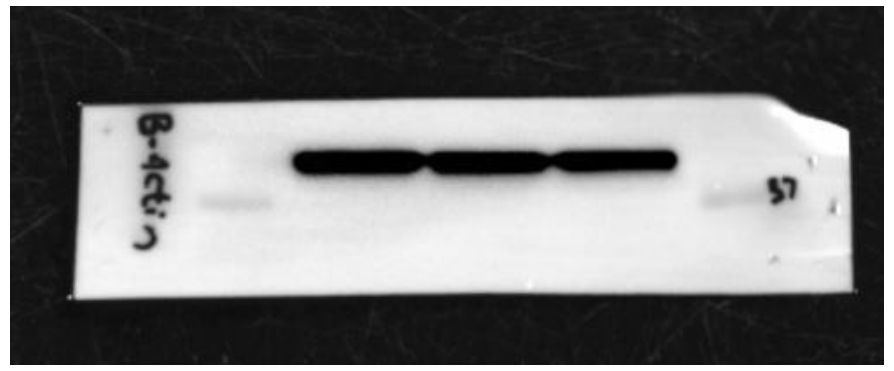

B-actin

Western blot images in Figure 4

Figure 4A

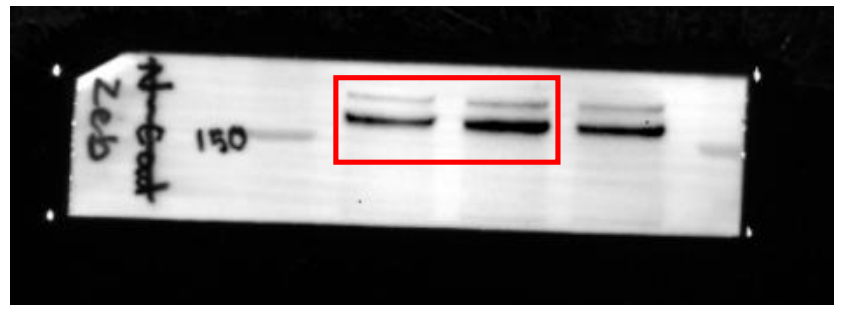

ZEB1

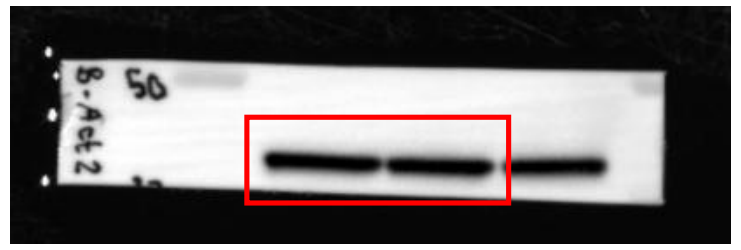

B-actin

Figure 4B

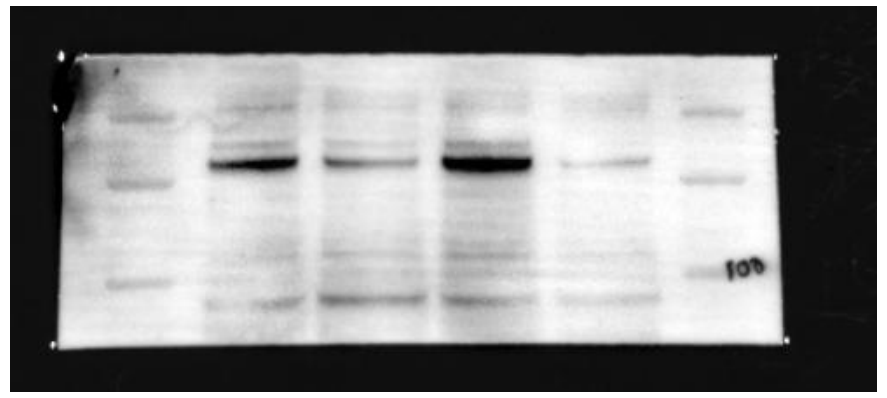

ZEB1

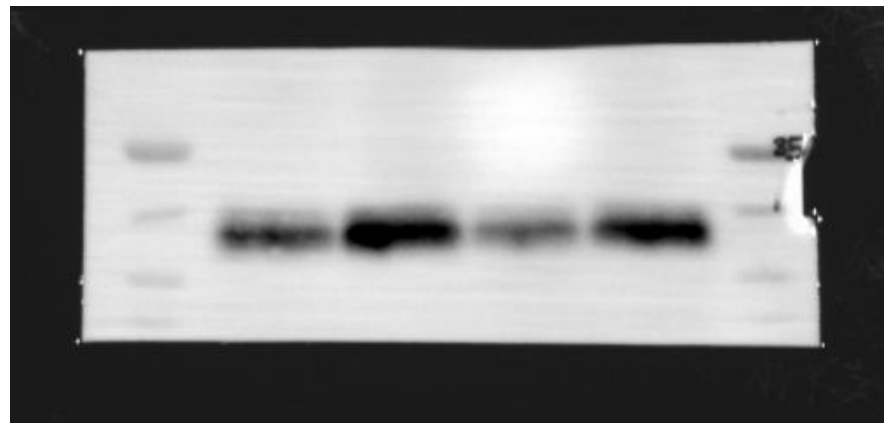

GPX4

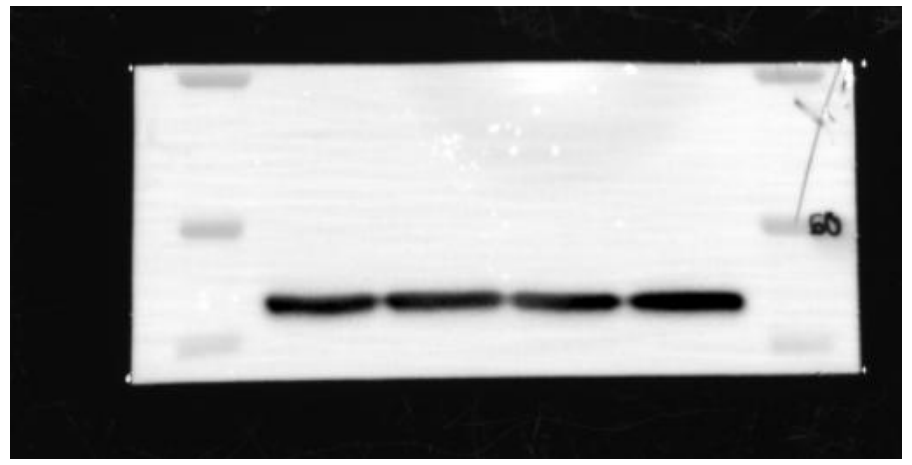

B-actin

Western blot images in Figure 5

Figure 5B

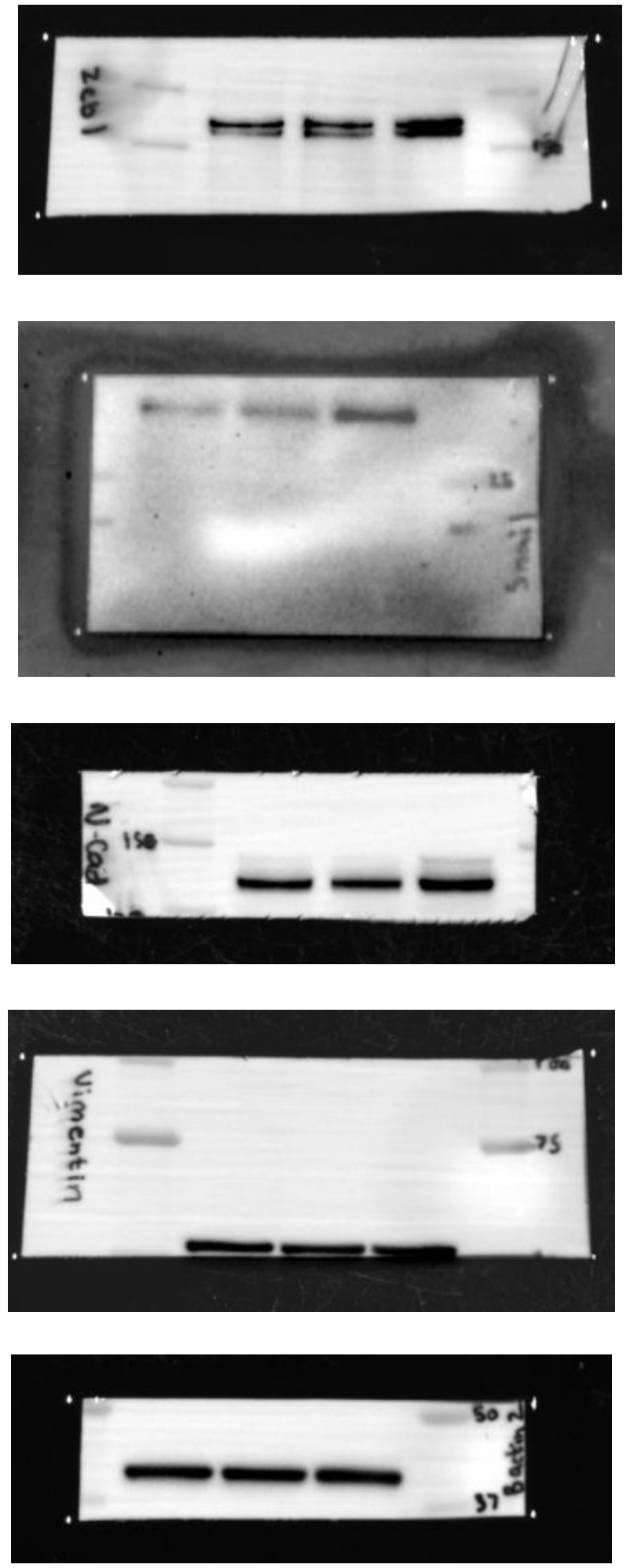

ZEB1

Snail

N-cadherin

Vimentin

B-actin

Figure 5C

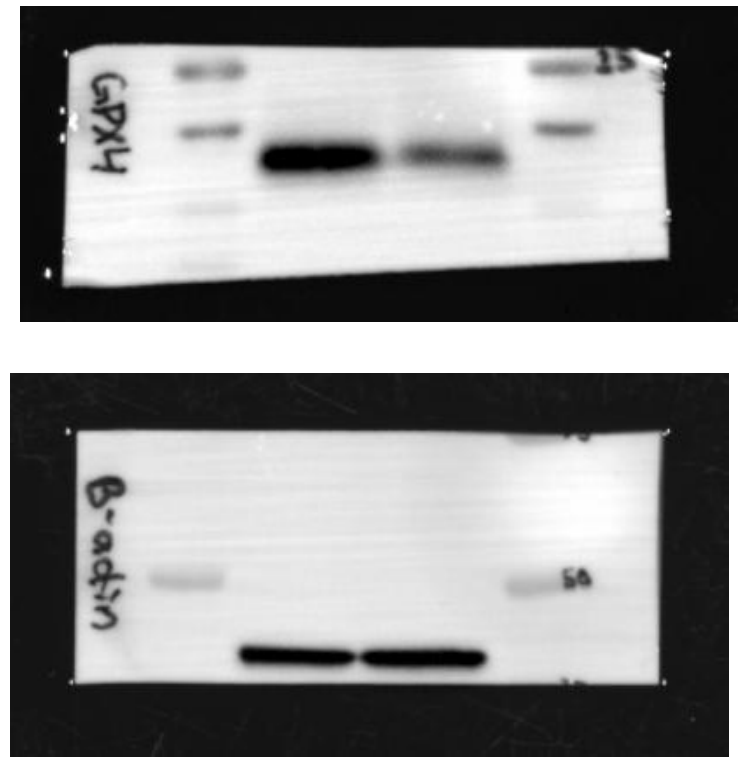

GPX4

B-actin

Western blot images in Figure 6

Figure 6E

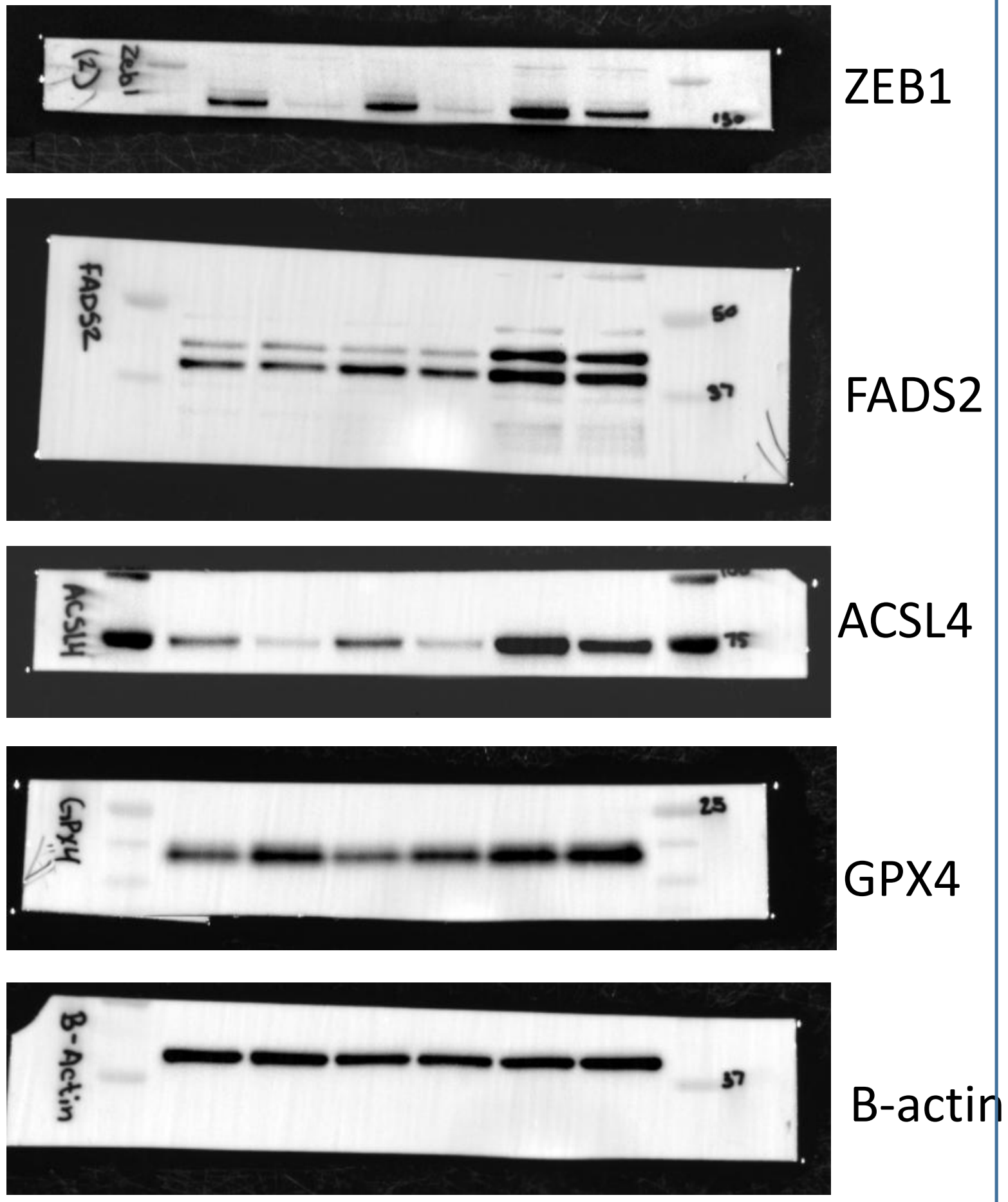

Figure 6H

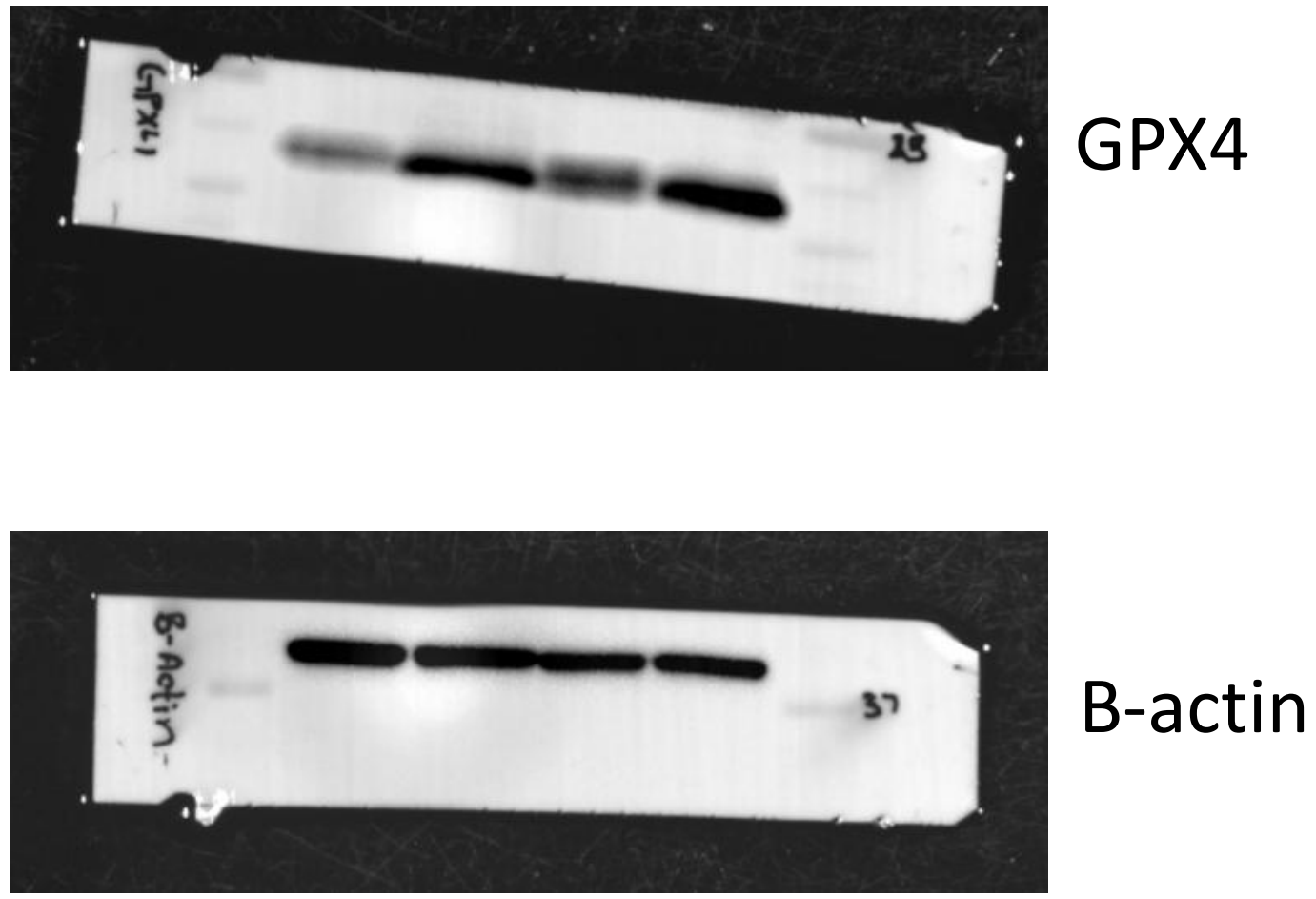

Figure 6I

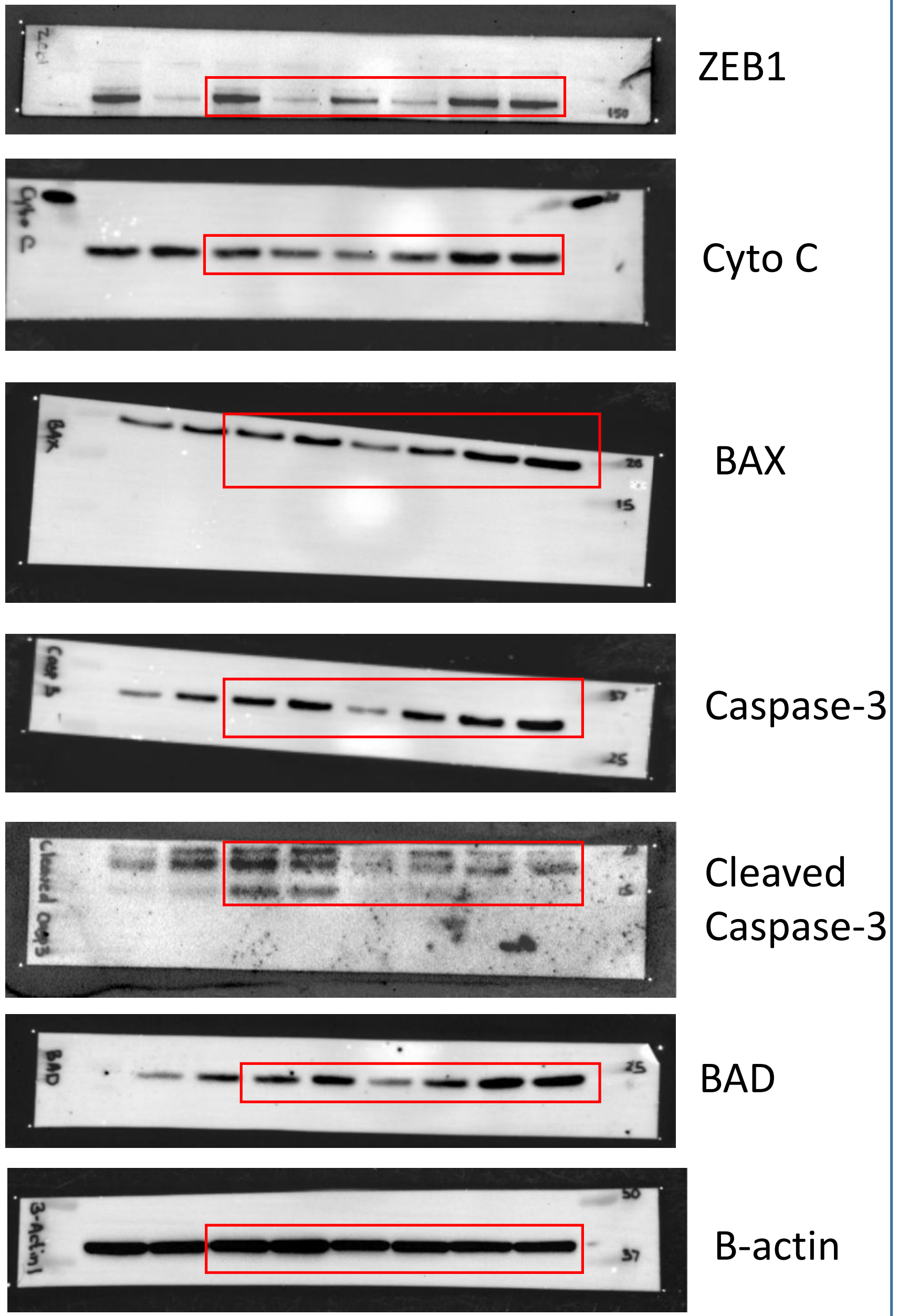

Agarose gel and Western blot images in Supplemental Figures 1B and 1C

Supplemental Figure 1B

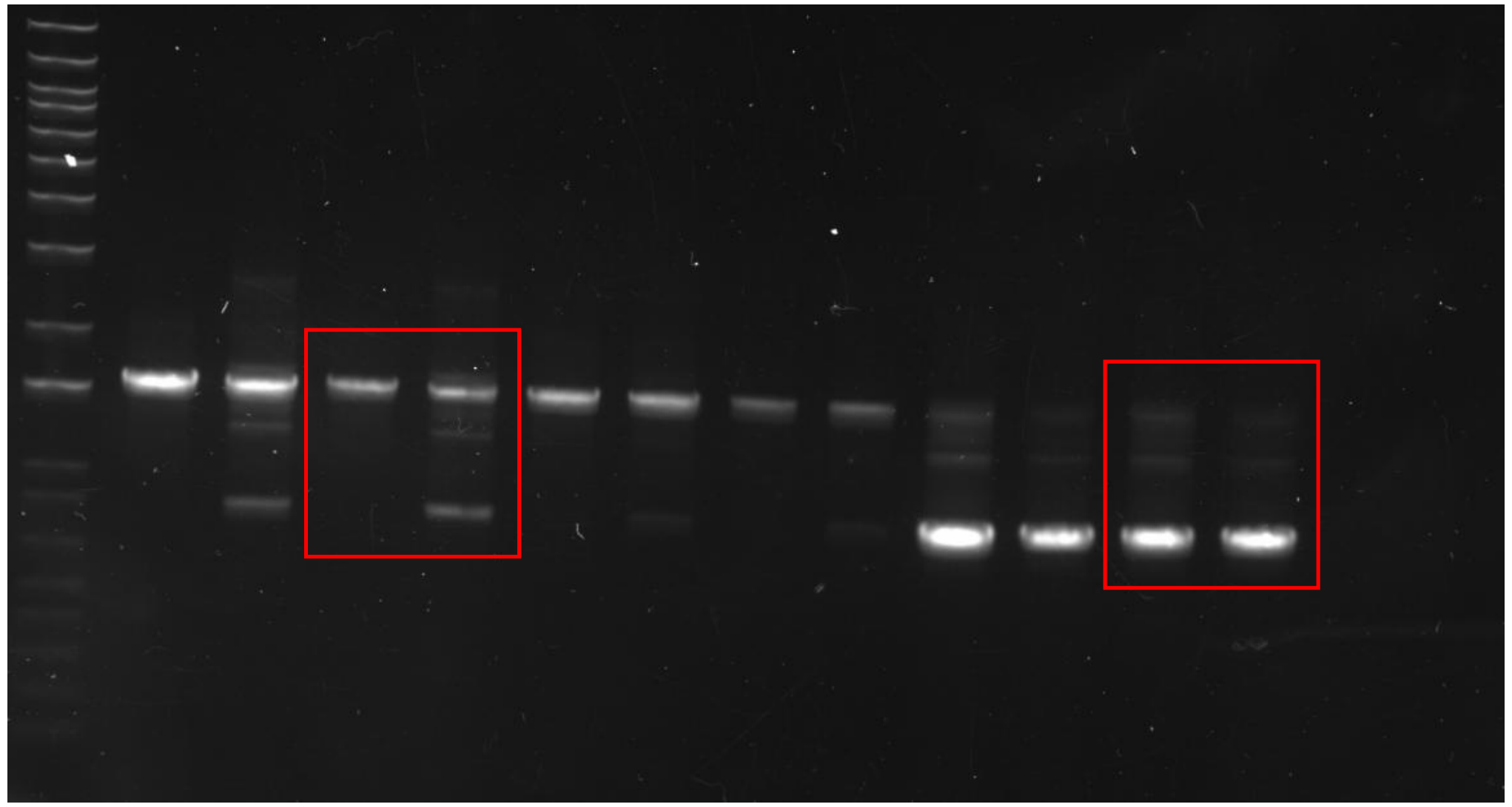

Supplemental Figure 1C

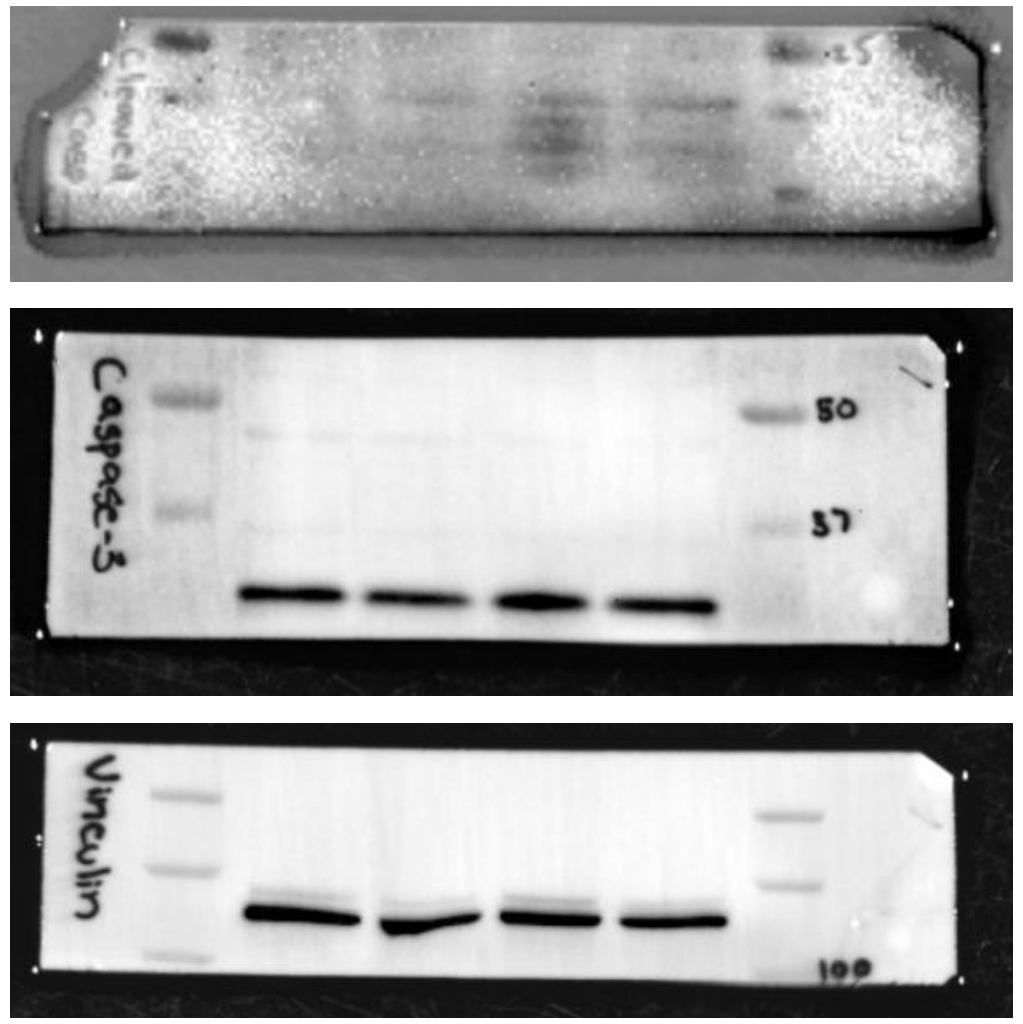

Western blot images in Supplemental Figure 5

Supplemental Figure 5A

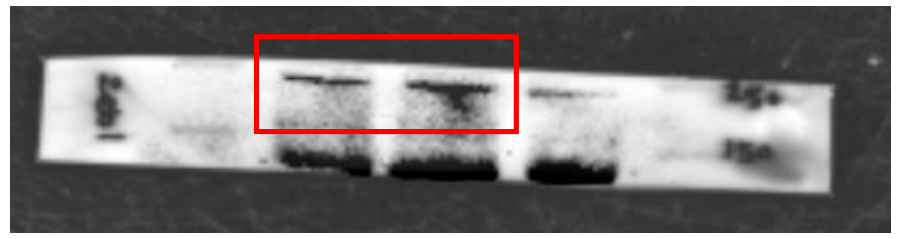

ZEB1

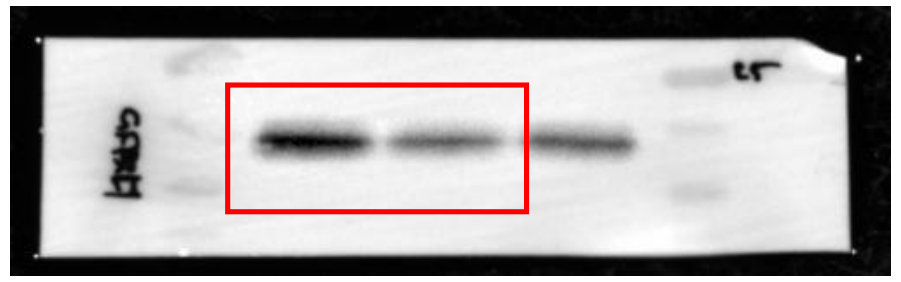

GPX4

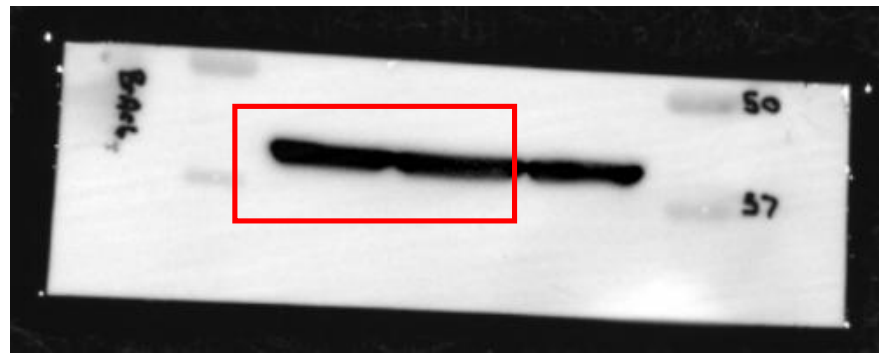

B-actin

Western blot images in Supplemental Figure 6

Supplemental Figure 6A

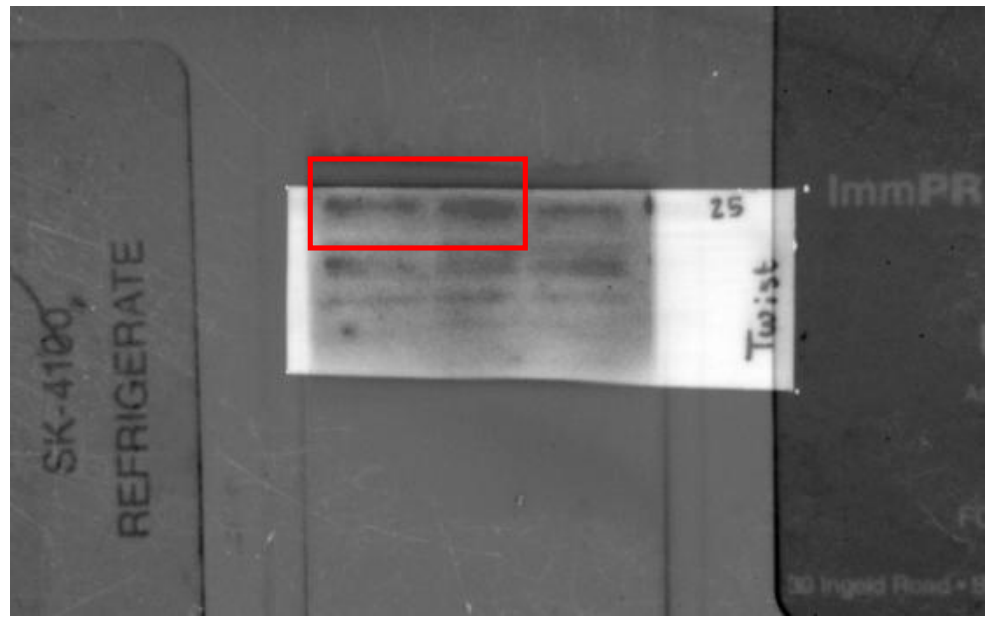

Twist
*Note: Ladder covered during chemiluminesent image to improve resolution

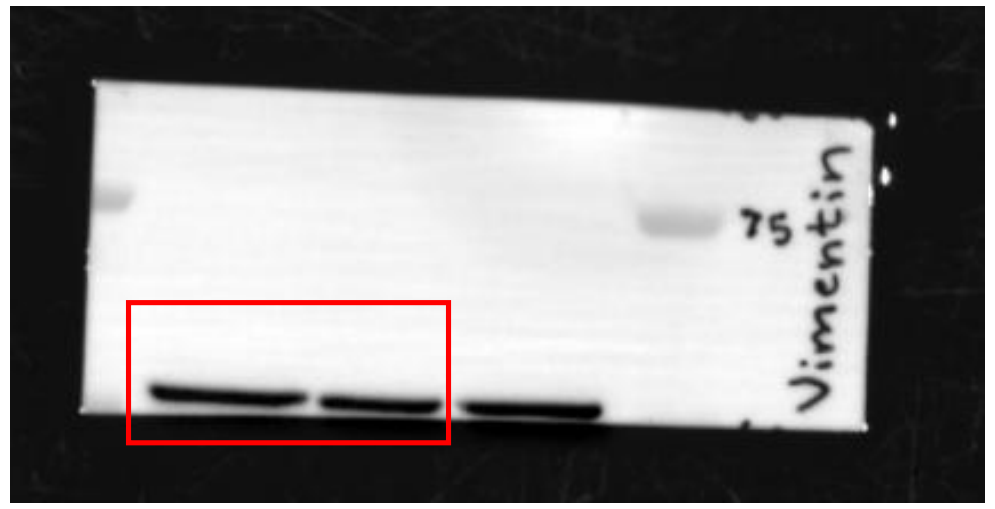

Vimentin

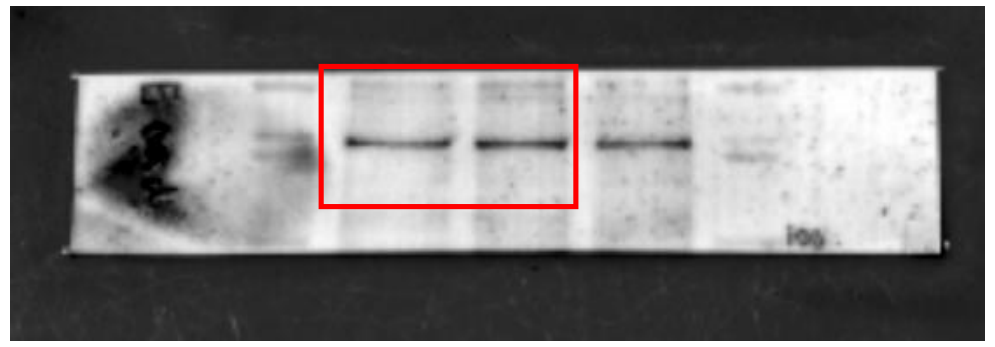

E-cadherin

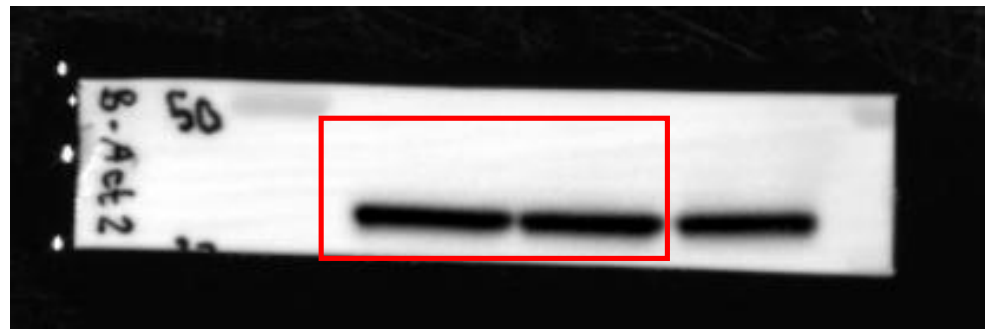

B-actin

Supplemental Figure 6B

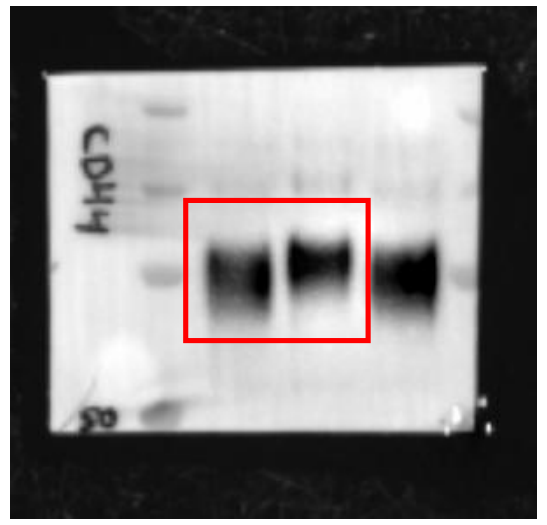

CD44

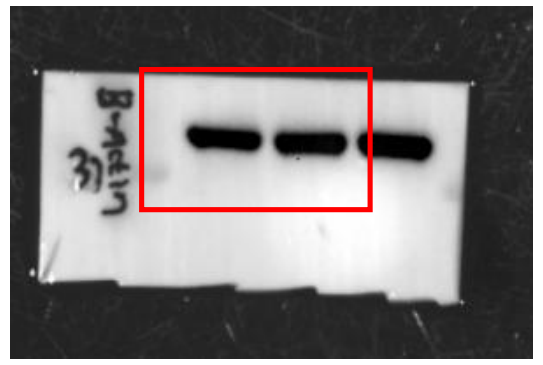

B-actin
